# Probiotic Potential of Traditional and Emerging Microbial Strains in Functional Foods: From Characterization to Applications and Health Benefits

**DOI:** 10.3390/microorganisms13112521

**Published:** 2025-11-02

**Authors:** Chijioke Christopher Uhegwu, Christian Kosisochukwu Anumudu

**Affiliations:** 1 Microbiology Unit, Bioscience Department, Federal University Otuoke, Otuoke 562103, Nigeria; chijiokeuhegwu@gmail.com; 2 Bioinformatics and Genomics Research Unit, Genomac Institute, Ogbomosho 210213, Nigeria; 3 School of Chemical Engineering, University of Birmingham, Birmingham B15 2TT, UK

**Keywords:** probiotics, functional foods, emerging microbial strains, fermented foods, gut microbiome

## Abstract

Global consumer demand for probiotic-enriched functional foods has increased as consumers become increasingly aware of the connection between what they eat and its role in their long-term health. Compared with conventional foods that primarily deliver fundamental nutrients, functional foods include biologically active compounds capable of influencing physiological processes. While traditionally used probiotic strains like *Lactobacillus* and *Bifidobacterium* are still at the center of this trend, there is growing interest in the exploration of emerging and novel microbial candidates that harbor new functional properties. This review addresses the characterization, modes of action, technological limitations, regulatory guidelines, and prospective health benefits of new probiotic strains in functional foods. The review further highlights the need for precise strain selection, novel encapsulation technologies for viability, and strict safety assessments in accordance with EFSA’s QPS (Qualified Presumption of Safety) and the United States FDA GRAS (Generally Recognized As Safe) specifications. Current research focuses on the classical benefits of probiotics, including gut microbiota modulation, immunomodulation, antimicrobial activity, lowering of cholesterol, and mental health. However, long-term clinical validation, strain specificity, personalized application, and effective communication to consumers are some areas where gaps remain. Addressing these challenges through the incorporation of omics technologies, synthetic biology, and more detailed microbiome–host interaction studies will be the key to unlocking the full potential of next-generation probiotics and sustaining consumer trust in this emerging market.

## 1. Introduction

### 1.1. Background

Functional foods are foods that have been scientifically established to deliver a positive impact on one or more specific physiological functions in the human body, other than simply providing nutritional benefits. These impacts can be towards considerably enhanced health, welfare, or lower risk of developing certain diseases [1]. With the increasing public awareness of the link between nutrition and health, there is more global momentum toward the use of food in disease prevention and health promotion. Consumers are also becoming more interested in dietary products that promote immune function, gastrointestinal health, and overall well-being. This is especially seen in the growing popularity of functional foods and dietary supplements enriched with bioactive components [2]. The concept of functional foods initially gained traction in Japan in the 1980s and was formally established by the Japanese Ministry of Health and Welfare in 1991 through the Foods for Specified Health Uses (FOSHU) system [3]. The system created an effective legal pathway for foods to carry approved health claims, provided they are backed by robust scientific and clinical evidence. Since then, the concept has expanded globally as consumers increasingly seek food products that actively support their health and lifestyle goals. Since diet is considered to be closely linked to several disease conditions and significant age-related chronic disease states, researchers and the food industry have turned their attention to functional foods that can provide more than calories and nutrients but also bioactive compounds with proven health impacts [1,4]. Similarly, consumers are increasingly becoming aware of the connection between what they eat and their long-term health conditions. Thus, many consumers are shifting from conventional foods that primarily deliver fundamental nutrients such as carbohydrates, proteins, fats, vitamins, and minerals to functional foods which contain biologically active compounds capable of influencing physiological processes such as probiotic yogurts, beverages with supplemented plant sterols, or prebiotic fibers. These foods have been found to contain vitamins, minerals, dietary fiber, antioxidants, phytochemicals, and bioactive peptides which are beneficial to human health [5,6]. This has fueled more interest in the consumption of functional foods as they help in disease prevention, immunity, digestive health, and other targeted health goals [7]. This increasing demand for functional foods has given rise to a rapidly expanding market for these products and this continues to grow. The combined global market value of functional foods and beverages was approximately USD 258 billion in 2021 and is expected to rise to USD 530 billion by the year 2028 [8], indicating the vast potential and business opportunity for developing new products, bridging the gap between nutrition and well-being.

Of all the bioactive compounds used to enhance the health attributes of functional foods, probiotics are of utmost significance. Probiotics are live microorganisms including bacteria and yeast which are incorporated into functional foods for consumption or application on the body to provide some health benefits [9]. According to Hill et al. [10], probiotics must be “safe for their intended use” and have “defined contents, appropriate viable count at end of shelf life, and suitable evidence for health benefits.” The International Scientific Association of Probiotics and Prebiotics (ISAPP) reaffirmed these ideas in a policy statement in 2018 [11]. Globally, probiotics rank as the fastest emerging class of dietary functional food supplements, and a growing body of research continues to indicate their extensive benefits to health [12]. Their overall acceptability comes from their recognized role of maintaining digestive and immunological well-being. Currently, probiotics are consumed regularly not only in yogurt but also in cheeses, beverages, cereals, and other emerging food delivery vehicles [13]. These probiotics, especially bacteria have been demonstrated to have potential such as neutralizing toxins [14], enhancing sensorial appeal when microencapsulated [15], and having improved survival for transit through the hostile environment of the gastrointestinal tract [16]. Several approaches are employed in introducing and maintaining probiotics in foods. Newer fortification strategies have been implemented to improve the potential and usage of probiotics. They have been utilized as additives in green tea fortified with soy [17], as added fortifications with vitamin D in products for weight management [18], incorporated in fruit juices to increase their health promotion [19], or as prophylactic treatments for cancer [20]. The global sales of probiotic supplements has continued to rise and was estimated at USD 96 billion in 2020 [1].

While the strains of *Lactobacillus* and *Bifidobacterium* have traditionally been the most widely utilized probiotics based on their established safety and effectiveness, scientific advancements and consumer interest have increased the exploration of new microbial strains as potential probiotics from diverse sources, including plants, fruits, and fermented foods. Hence, this review provides an overview of the current state of emerging probiotics in functional foods, from strain selection and characterization to their mechanisms of action, regulatory considerations, technological challenges, and future perspectives for research and applications.

### 1.2. Sources of Novel and Conventional Probiotic Strains

Probiotic strains, whether well-known or novel, can be isolated from a wide range of natural or traditional sources. Over the years, several strains of yeasts and LAB with techno-functional properties have been isolated from ethnic beverages, unique sources such as plants, animal hosts, and marine ecosystems [21]. Table 1 summarizes different sources of both conventional and emerging probiotic candidates and their main functional features.

### 1.3. Conventional vs. Emerging Probiotic Strains

For several decades, the global probiotic industry has relied on a relatively small number of well-studied, widely accepted microbial species. Those relevant to the human gut microbiota among them are majorly members of the genera *Lactobacillus* (now reallocated to many new genera such as *Lactiplantibacillus*, *Ligilactobacillus*, *Lacticaseibacillus* and *Limosilactobacillus*) and *Bifidobacterium* [21,36]. These bacteria are naturally found in the human gut and fermented foods and have a long record of safe ingestion backed up by a strong body of scientific evidence [21,37]. Some of the common examples include strains of *Lactobacillus acidophilus*, *Lacticaseibacillus rhamnosus* GG, *Limosilactobacillus reuteri*, *Bifidobacterium bifidum*, *Bifidobacterium longum*, *Bifidobacterium animalis* subsp. *lactis*, etc. These strains have reportedly been characterized by survival under stomach acidic conditions, ability to colonize the intestinal epithelium, ability to compete with and inhibit pathogens thereby keeping the gut healthy, producing essential metabolites such as short-chain fatty acids, and modulating the host’s immune system [2,37]. Due to their proven efficacy and safety, they have been used over the years in different products such as in fermented foods, supplements, infant formula, and even as therapeutic treatments of gastrointestinal and immune diseases [21,31,38].

While milk and other dairy products continue to be the major vehicle for probiotics delivery to consumers, interest in their non-dairy probiotic food counterparts including cereals, fruits, and vegetables has expanded over the last few decades [39]. This is primarily due to changing food habits, i.e., the growing rate of vegetarianism and veganism, and the accelerated rate of milk allergy, cholesterol, and lactose intolerance which limits the consumption of dairy products by many [5]. Identification of new strains suitable for these foods and niche markets opens the door to more opportunities for functional foods innovation. However, it is noteworthy that most of the probiotic cultures used in food products commercially available today are derived from human gut microbiota or traditional dairy fermentations [21]. The dairy sector which is strongly associated with probiotics, is the largest functional food market, accounting for nearly 33% of the broad market, while cereal products have just over 22% [13]. For stable supplementation of probiotics into plant or cereal-based foods, strains that come directly from plants or plant fermentations tend to be better adapted to thrive. The use of strains well adapted to these substrates is more effective as non-dairy food matrices have particular technological, environmental and physiological challenges, that typical dairy or gut-derived probiotic strains would not be able to survive in optimally [40]. Thus, the optimization of novel probiotic strains to survive, be potent and viable in non-dairy food product matrixes is critical in the development of new functional foods that cater to different dietary needs. Technological advancements have made it feasible to modify food components in a regulated manner, such as modifying food components, among other methods, to change specific structural aspects of foods such as fruit and vegetables [41], making them conducive for probiotic supplementation. These foods are nutrient-rich and contain essential minerals, vitamins, dietary fibers, and antioxidants which makes them perfect substrates for probiotics culture [21]. The idea of using fewer milk components as vehicles of probiotic agents or even substituting milk with other media, like cereals, fruits, and vegetables, is also encouraged by traditions and financial considerations that restrict the use of dairy products due to religious, raw material unavailability, and other practical reasons [13]. Hence, it has become crucial to leverage the numerous advancements in scientific innovation and enhanced screening technologies like genomics, metagenomics, innovative culturing techniques and metabolomics, to optimize the customization of probiotic products using newer strains and food matrices for specific nutritional needs, disease prevention, and even adjunct therapy for conditions like obesity, diabetes, and inflammatory bowel disease. To provide a clearer perspective of the current frontiers of probiotic research, a conceptual comparison between conventional, well-established probiotics and newly emerging candidates that exhibit probiotic-like properties but are still under safety and regulatory evaluation is presented herein (Table 2).

## 2. Biological and Functional Characteristics of Probiotic Strains

**Figure 1:**
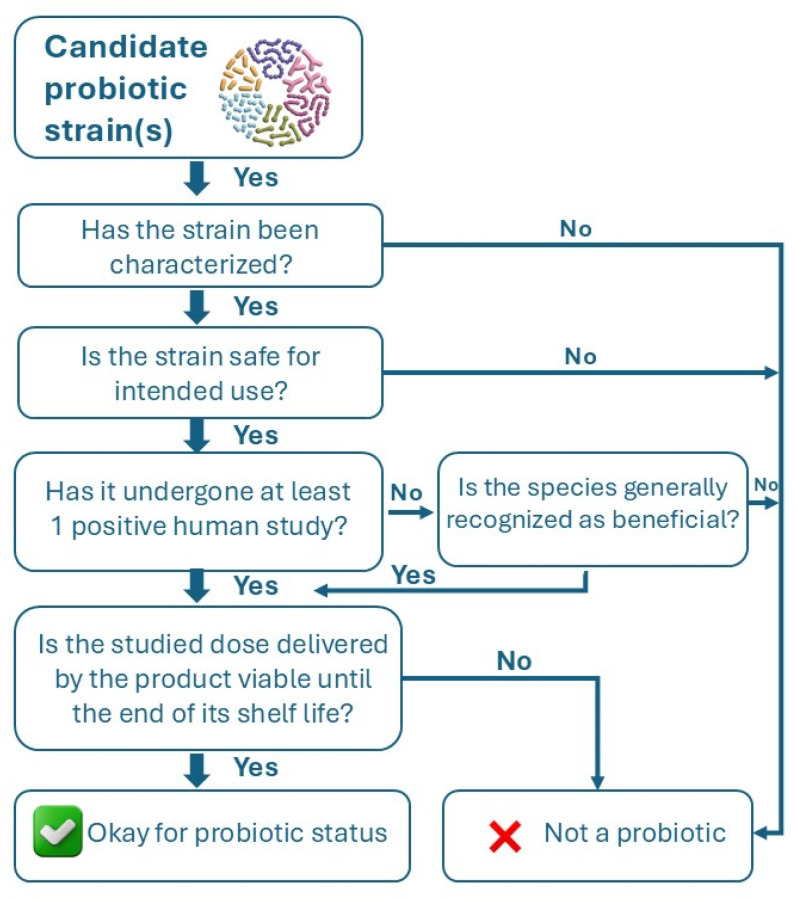
Decision tree used to assess whether a potential probiotic meets the requirements (Adapted from Binda et al. [2]).

For new probiotic strains to be validated, a rigorous scientific framework is required to ensure safety, efficacy, and viability [2]. Several international organizations, most notably the Food and Agriculture Organization (FAO) and the World Health Organization (WHO), have provided globally accepted definitions and guidelines for the classification and characterization of specific strains of microorganisms to qualify as a probiotic for use in foods and dietary supplements. These criteria include that probiotic strains must be (i) adequately characterized; (ii) safe for the intended use; (iii) supported by at least one positive human clinical trial; and (iv) alive at an efficacious dose in the product throughout shelf life [46]. As presented in Figure 1, these criteria can serve as a useful tool in determining whether a candidate strain, or combination of strains, can serve as probiotics or not.

**Figure 2:**
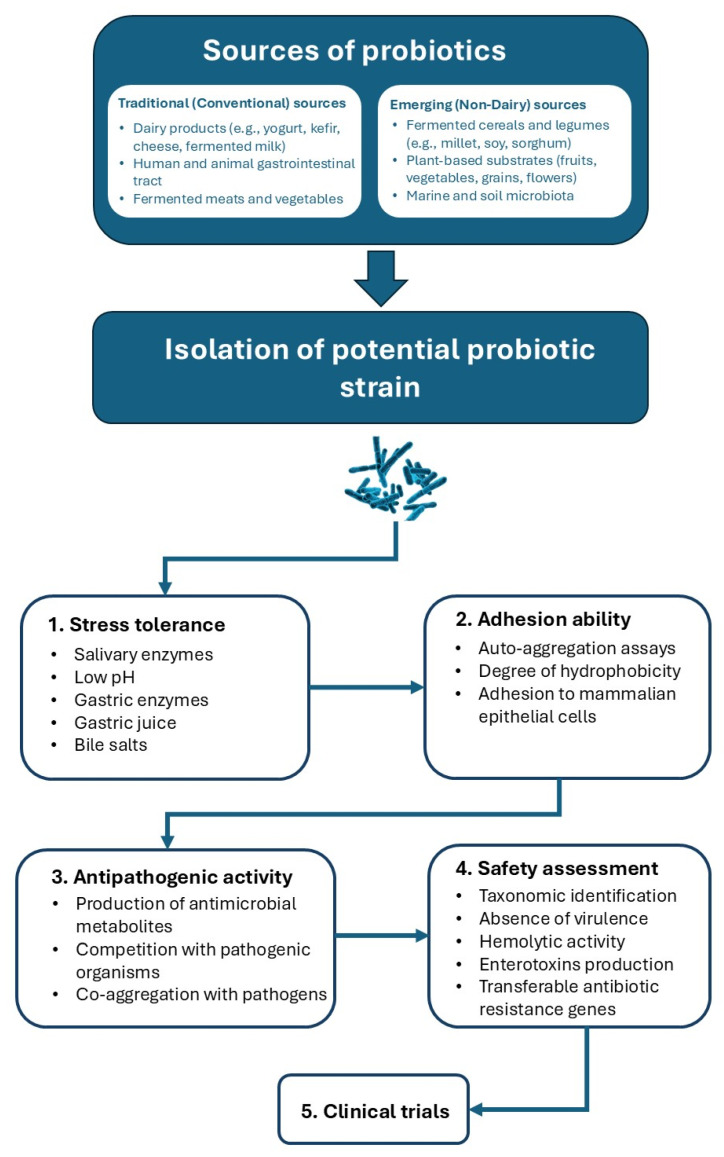
The step-by-step screening approach used for characterization of probiotic strains according to WHO/FAO guidelines.

The lactic acid bacteria (LAB) that are mostly used as probiotics are mainly species of *Lactobacillus* (*L. acidophilus*), *Lacticaseibacillus* (*L. rhamnosus* and *L. casei*), *Lactiplantibacillus* (*L. plantarum*), *Ligilacto bacillus* (*L. salivarius*), and *Limosilactobacillus* (*L. reuteri* and *L. fermentum*). Furthermore, strains of the *Bifidobacterium* genus (*B. longum*, *B. bifidum*, *B. lactis*, *B. animalis*, *B. breve*, and *B. infantis*) and yeasts (*Saccharomyces cerevisiae*) are commonly used [21]. Probiotic strains are majorly selected, putting into consideration several aspects such as the consumer’s safety (hemolytic activity, mucin degradation, and antibiotic susceptibility), physiological functionalities (bile salt and acid tolerance, auto-aggregation, bile salt deconjugation, co-aggregation with pathogens, cell surface hydrophobicity, ability to survive when exposed to gastrointestinal conditions and antagonistic action against pathogens) [21]. Furthermore, technological aspects such as tolerance to sodium chloride (NaCl), production of bioactive compounds, and proteolytic and lipolytic activity are also taken into consideration [47]. The “step-by-step screening approach” as shown in Figure 2 and discussed in the following sub-sections is majorly followed for the selection of potential probiotic microorganisms isolated from traditional and emerging sources. The strains are generally subjected to a series of tests to determine their characteristics. The strains that exhibit the greatest number of functional characteristics and no undesirable traits are chosen at the conclusion of this process. Additionally, proper strain identification and typing are essential because many probiotic attributes are strain specific. Even within the same species, different strains can vary in their ability to survive gastrointestinal stress, adhere to intestinal cells, or exert antipathogenic effects. Hence, strain-level identification using genetic and molecular methods such as 16S rRNA sequencing or whole-genome sequencing (WGS) is crucial for accurate probiotic characterization and safety assurance [2,48].

### 2.1. Ability to Tolerate Stress

The ability to withstand the variety of stress conditions associated with food processing and the human GIT is a crucial test in the selection of food-grade emerging microbial strains. A potential probiotic strain must demonstrate resilience from the moment it is swallowed until it is effective in the gut before it can be considered acceptable for consumption as a probiotic in foods or dietary supplements. Upon ingestion, probiotics must survive enzymes naturally present in the mouth, such as amylase and lysozyme. While most Gram-positive bacteria are vulnerable to the activity of lysozyme, many strains of LAB have demonstrated significant resistance lysozyme, and can thus, survive as part of the human oral microbiota [49,50]. Once swallowed, the probiotic must withstand harsh conditions in the gastrointestinal tract, which involves survival of the highly acidic stomach environment, digestive enzymes like pepsin, and later, bile salts and pancreatic juices in the small intestine [50]. The probiotic must also withstand mild heat stress from the body temperature. To survive these conditions, candidate strains must possess good acid and bile salt tolerance or mechanisms to neutralize or expel such inhibitory agents [2]. For probiotics, tolerance to bile and acidity is necessary for their survival in the upper digestive system and passage to the large intestine. Thus, a potential probiotic’s survival in the gastrointestinal tract is predicted by its ability to survive in simulated gastrointestinal settings [51]. For confirmation of these survival properties, possible strains are cultivated in media containing different pH conditions and stress elements such as pepsin, lysozyme, amylase, bile salts, NaCl, Oxgall, and porcine pancreatic or gastric juices added. Their viability is then determined by quantifying viable cell numbers or growth over time [50].

Studies have demonstrated that different isolated microorganisms with potential probiotic properties are able to survive simulated gastrointestinal conditions. For instance, Garcia et al. [52] evaluated five selected *Lactobacillus* strains (*L. brevis* 59, *L. pentosus* 129, *L. paracasei* 108, *L. plantarum* 49, and *L. fermentum* 111) isolated from byproducts of fruit pulp processing for desirable probiotic-related properties and found that exposure to pH 5.0 and to bile salts (0.15, 0.30, and 1.00%) to simulate gastrointestinal conditions did not decrease the counts of the *Lactobacillus* strains. Results from their study also suggest that acid and bile salt tolerance are generally strain-specific, indicating that some strains may not withstand the harsh conditions of the gastrointestinal tract [52]. However, gastrointestinal stress resistance varies greatly across species and strains and only a few isolated strains pass these rigorous screening tests which have been confirmed by different experimental studies. For example, out of the fifteen potential probiotic yeast strains isolated from pistachio fruits (*Pistacia vera*) in the study by Fernández-Pacheco et al. [53], only 65% of them had a high survival rate in the gastrointestinal conditions they were tested in. In another study by Maragkoudakis et al. [54], they found that out of 29 *Lactobacillus* strains tested, only six survived when pH 1 was applied for an hour, and eight strains could not survive pepsin at pH 2. Generally, pH values ranging from 2 to 5 and bile salts concentrations from 0.3 to 2% are considered critical selection limits for potential probiotic microorganisms [21,55].

### 2.2. Adhesion Ability

It is essential to determine the potential of emerging probiotic strains to adhere to the gastrointestinal epithelial cells. Effective adhesion is a functionally significant attribute since it allows the probiotic to transiently colonize the gut mucosa, produce biofilms, and exhibit actions like competitive exclusion of harmful microorganisms, modulation of the immune response, and interaction with host tissues [50]. The adhesion is a complex molecular interaction between the contact and binding of two biological membranes (the probiotic cell surface and the host gut epithelial cells). The physicochemical characteristics of microbial cell surfaces play a major role in determining this interaction. Specifically, the extent, strength and selectivity with which a probiotic strain adheres to mucosal surfaces is influenced by surface proteins, exopolysaccharides, and other extracellular structures [56,57]. Microbe adhesion to epithelial cells is further influenced by the cell surface’s hydrophobic characteristics as well as its ability to auto-aggregate. While cell surface hydrophobicity enables a better connection between microbes and human epithelial cells, microbial cell auto-aggregation allows for the probiotic cells to clump together in flocs, which allows them to be more locally dense in the gut and adhere and colonize epithelial surfaces better [50]. The process can be measured by a simple test where the strain is added to phosphate-buffered saline (PBS) solution, and absorbance changes are tracked with time to see how well the cells aggregate together [55,58]. Cell surface hydrophobicity is another parameter of a strain’s attachment capability. Hydrophobic cell surfaces are readily interlocked with human epithelial cell membranes, consisting of high lipid concentrations, for superior contact and firm attachment. This is measured on a routine basis by the Microbial Adhesion to Hydrocarbon (MATH) test which involves mixing the microbial suspension with a hydrocarbon phase (e.g., hexadecane or xylene) and water [57,59]. The hydrophobicity of the strain is then determined by the degree of bacterial affinity for the hydrocarbon phase, expressed as absorbance at 600 nm [50]. Finally, direct adhesion assays with mammalian epithelial cell lines such as Caco-2, HT-29, or fetal I-407 cells are also widely used to ascertain the actual binding capacity of a strain [60,61].

Furthermore, the colonization of the gut by probiotics is usually preceded by adhesion to the intestinal mucosa leading to the formation of biofilms, which does not favor the binding of enteropathogens [43]. Probiotics isolated from traditional fermented foods have been shown to demonstrate adhesion properties which were linked to the synthesis of exopolysaccharides and enzymes that encourage interactions between probiotics and host-specific receptors. Furthermore, they function as a cell surface capsule during food preservation or during gastrointestinal tract passage, shielding the probiotics against harmful substances and stressful situations [21,31,38].

### 2.3. Antipathogenic Activity

For effective probiotic activity within the gut, following successful microbial adherence to the intestinal lining, probiotics can synthesize a range of antimicrobial compounds/bioactive metabolites which aids in host defense by engaging in direct antagonistic activities against pathogenic microorganisms [50]. This protective effect is a key factor when evaluating the performance of emerging microbial strains for safe and effective use in functional food. Probiotics synthesize these bioactive compounds by metabolizing available carbohydrates, proteins, and other dietary components into bioactive metabolites that inhibit or kill pathogens. These include the production of organic acids (which reduce local pH to create an unfavorable environment for most pathogenic microbes), digestive enzymes, hydrogen peroxide, bacteriocins, and other low-molecular-weight antimicrobial peptides [62]. Combined, these metabolites inhibit the growth and activity of pathogenic bacteria in the gut. In addition, probiotics also participate in other antagonistic activities such as direct competition with pathogens for nutrients, physical deterrence of occupation of attachment sites by pathogens on the gut epithelium, and even coaggregation with them, forming clumps that traps pathogens, thus, allowing for their expedited removal from the gut by normal digestion and excretion [22,29]. LAB strains such as *L. plantarum* 53, *L. fermentum* 60 and 296, and *L. paracasei* 106, isolated from fruit by-products, were found to demonstrate strong auto-aggregation abilities which enhanced their ability to create physical barriers in the gut, inhibiting the colonization of pathogenic microbes through the occupation of adhesion sites and the inhibition of their access to epithelial surfaces [47].

Interestingly, these beneficial activities are highly strain-specific. Species and strains vary considerably in the types and quantity of antimicrobial substances produced by them and in their effectiveness at competing-out or aggregating-out pathogenic bacteria [63]. To determine these traits in a candidate strain, basic laboratory assays including the agar inhibition zone assay is undertaken to measure the antimicrobial activity by observing clear zones where pathogen growth is restricted [64]. Furthermore, competition can be investigated by testing the capacity of a probiotic to interfere with pathogen adhesion to intestinal cell lines in vitro [65]. Similarly, capacity for coaggregation is assessed by co-cultivating the probiotic with common pathogens like *E. coli*, *S. aureus*, *Candida* spp., or *L. monocytogenes*, and noting whether the probiotic will bind to and aggregate the pathogens, which will enable their expulsion from the gut [29,66].

The beneficial health-promoting potential of probiotic strains are directly related to these distinct biological properties, which allow them to adapt effectively to the body systems of the host and the gut microbiota [37]. The ability of probiotics to modulate the immune system is one of the primary mechanisms by which they achieve their positive impacts. Certain strains can boost the immune response of the host, making it easier for the body to defend itself against diseases and infection [37]. In addition, probiotics also play a significant role in neutralizing colonization of pathogenic microorganisms by competing for adhesion sites and nutrients in the gut. This defense mechanism is complemented by the production of various antimicrobial compounds, including organic acids (e.g., lactic acid), hydrogen peroxide, lysozymes, and bacteriocins [62]. Bacteriocins for instance, are peptide-based compounds with high potency and low toxicity, usually produced in situ by probiotics as an adaptation mechanism, and can exert a bactericidal or bacteriostatic effect against closely related strains of the producer organism and other bacteria genera [62]. These antimicrobial agents support the equilibrium of the gut environment by lowering the pH and preventing harmful microbes from colonizing the gut. Additionally, probiotics can synthesize basic digestive enzymes such as amylase, protease, lipase, pectinase, and endoglucanase, beneficial for the breakdown of complex food constituents, making nutrients more accessible and easier to digest. Most probiotics also synthesize vitamins, such as vitamin A, some B-group vitamins (e.g., B1, B2, B6, B12), C, E, K, PP (niacin), beneficial for the nutrition and health of the host [37].

### 2.4. Safety and Validation Assessment

In introducing live microbial strains into diets to be consumed by humans, especially from unconventional sources, safety assessment is a priority. Although probiotics are deemed safe, for new probiotic strains, they have to undergo stringent, evidence-based examination to confirm safety [2]. For the safety of consumers, some legislations worldwide such as the European Union Novel Food Regulation, Qualified Presumption of Safety (QPS) list, PROSAFE initiative, and U.S. FDA, WHO, and Canada’s NHPR have established strict standards for probiotic safety assessment for use in humans. The standards typically require extensive documentation of the strain’s origin and history of isolation; conclusive taxonomic classification; and proof that the strain is devoid of virulence determinants, infectivity, toxins, or antibiotic resistance gene transferability [50,67].

Traditionally, most probiotic strains that are used in commercial products were sourced from healthy human beings. This approach ensures the highest likelihood that the microbes are best adapted to survive and work within the human gut [2,10]. However, with ongoing efforts to identify new or emerging probiotic strains in unorthodox sources such as fermented foods, plants, marine environments, or soil, rigorous safety screening is now more essential. For any novel isolated strain, early and precise identification is a critical step in the selection process. This initial identification helps in eliminating any strains that possess pathogenic or undesirable traits [68]. Primary species-level distinction tends to combine classical biochemical tests such as metabolic profile and ability to grow on a variety of carbon sources, Gram stain, catalase, nitrate reductase, and urease tests with genetic methods like 16S rRNA gene sequencing to validate taxonomic allocation [50]. However, for the sake of ensuring probiotic safety and traceability at the strain level, such common techniques are not sufficient on their own because they might not be able to offer adequate genetic variability to differentiate between strains that are nearly related. Therefore, molecular fingerprinting techniques such as Repetitive Element Palindromic PCR (rep-PCR), Random Amplified Polymorphic DNA (RAPD), or Pulsed-Field Gel Electrophoresis (PFGE) are usually utilized in order to ensure precise strain-level discrimination [69,70]. While previous probiotic research has focused on routine safety testing like enterotoxin testing or hemolysis testing, the requirements now are more rigorous, with comprehensive safety screening of any emerging strain intended for human consumption. Beyond screening for basic characteristics, it has become essential to screen for other risk factors such as the production of undesirable metabolites like D(-)-lactate, deconjugation of bile salts, and virulence factors like gelatinase, DNAse, or hemolysins. Identification of such possible hazards relies on molecular techniques, such as PCR-based identification of virulence genes, cytotoxicity assays, and, where required, confirmatory in vivo testing [26,43,71].

One of the key issues with the safety of modern probiotics is the assessment of antibiotic resistance potential, as this has become a high priority [2]. This is because there is a risk for probiotics to pass on resistance genes to gut pathogenic bacteria through horizontal gene transfer, which would be an issue for public health [36]. Accordingly, QPS and GRAS evaluations now explicitly include checks for resistance traits [72]. Resistance in probiotic strains are screened by methods such as agar disk diffusion, E-tests, or minimum inhibitory concentration (MIC) assays that determine the minimum dose of an antibiotic to inhibit bacterial growth [73]. For LAB, specific media like LAB susceptibility test medium (LSM) are used to avoid deceptively false results by media incompatibilities during antibiotic susceptibility tests [74]. However, these are phenotypic tests and are not standardized between laboratories. Therefore, PCR has emerged as the method of choice for reproducibly detecting antibiotic resistance genes because of its sensitivity and reliability. Using PCR, DNA for probiotic strains is amplified with gene-specific primers, and products may be confirmed by sequencing to be assured of gene identity [50].

Interestingly, not all antibiotic resistance poses equal risk. If a probiotic’s resistance is intrinsic, i.e., it is chromosomally mediated and not on transmissible elements, then there is little risk of passing it onto other gut bacteria. In such a case, resistant probiotics are also effective in restoring the microbiota after treatment with antibiotics. However, horizontally transferable acquired resistance genes on plasmids or transposons involve a significant risk of transfer and must be rigorously tested [36,72]. Filter mating experiments, in which a probiotic with a detected antibiotic resistance gene is cultured with recipient cells that do not have the gene, can be used to analyze gene transfer. Phenotypic and molecular methods can then be used to analyze the transfer ratio to the recipient cell [75]. In recent times, WGS which includes all extrachromosomal components, has been employed as a powerful and cost-effective “gold standard” approach for strain identification and screening of antibiotic resistance genes in probiotic candidates. Through full sequencing and mapping against available reference databases, such as the National Center for Biotechnology Information (NCBI), WGS enables identification of microbes down to the species and strain level and helps determine the likelihood of resistance gene transmission to other gut microbes [2,76].

Finally, beyond genomic and toxicological evaluations, the ultimate validation of probiotic safety and efficacy requires human clinical evidence. For a candidate microbial strain to qualify and be granted probiotic status, it must demonstrate a measurable health benefit in at least one well-designed human clinical trial, ideally followed by confirmation studies [2]. Randomized, placebo-controlled, and double-blind trial designs, compliant with recognized clinical research standards such as The Consolidated Standards of Reporting Trials (CONSORT), are strongly recommended to ensure reliability and reproducibility of outcomes. These trials help confirm not only the strain’s tolerability and absence of adverse effects but also its ability to confer the intended physiological benefit in humans [77,78]. In addition to clinical safety, probiotics must remain alive and at an efficacious dose throughout product shelf life [46]. While no fixed dose is included in the formal definition of probiotics, probiotics must be taken in a quantity sufficient to provide a health effect. Although some microbes can exert effects at low dosages due to their capacity to replicate within the host, probiotics generally require sufficient viable counts to provide beneficial effects [10]. Unlike pharmaceutical agents, probiotics are living organisms, and dose-ranging or maximum tolerable dose (MTD) studies are rarely conducted. This is majorly due to the notion that food ingredients are presumed safe [2]. Given the general presumed safety profile of probiotics and the fact that they are mostly not subjected to MTD or dose ranging studies, most studies and products use doses ranging from 10^8^ to 10^11^ colony-forming units (CFU), based on evidence from previous trials demonstrating efficacy and safety [2]. Finally, to ensure reliability and consistency, probiotic viability must be measured using standardized enumeration methods, such as CFU plating on selective media or flow cytometry [79]. These methods help confirm that the stated dose is maintained during clinical studies and that the probiotics remain viable and effective throughout a product’s shelf-life. Ensuring this stability and viability is essential for both consumer safety and regulatory compliance.

## 3. Emerging Probiotics

Traditionally, probiotics have been derived from well-established genera like *Lactobacillus* and *Bifidobacterium*. However, the growing demand for evidence-based functional foods and alternatives suitable for lactose-intolerant individuals has driven interest in novel probiotic strains from non-dairy sources such as traditional fermented drinks, raw vegetables, fruits, and flowers [21,80]. Hence, in the last decade, these unconventional sources have been actively explored for microbial strains with both techno-functional properties and probiotic potential, offering promising opportunities for biotechnological applications and/or functionalization of foods.

### 3.1. Emerging Probiotics from Non-Dairy Fermented Foods

In recent times, emerging probiotics have frequently been isolated from non-dairy and unconventional sources such as traditional fermented foods, made with a broad range of raw materials of both plant and animal origins. These foods reflect rich culinary and cultural traditions, which are often passed on across generations and modified to suit local raw materials availability in different regions of the world. The microbial composition of such foods is determined by the type of raw material used and, consequently, the type of probiotic species and strains that may be isolated from them [80]. Non-dairy fermented foods, (especially those of vegetable, meat, and seafood origin), have proven to be rich sources of LAB and other beneficial microorganisms. For instance, LAB have been easily recovered from fermented fish products, cured or dried meats, fermented meats, salted crabs, and other seafood products [81,82]. Additionally, plant fermentations centered on substrates such as soybeans and vegetables (e.g., cabbage, carrots, and mustard greens) also harbor dense microbial populations, which are often dominated by LAB such as *Lactobacillus*, *Leuconostoc*, *Pediococcus*, and *Weissella* species.

LAB in traditional fermented foods vary depending on the substrate and fermentation environment. For instance, in fermented fish and crustacean products, *Enterococcus* is the dominant LAB genus present and is usually characterized by its high tolerance to salt [80]. On the other hand, *Lactobacillus* species are more commonly isolated from fermented meats and vegetable products. These microbial tendencies are due to adaptation to the salinity of the environment where fermentation occurs. The halotolerance of LAB strains from species such as *Enterococcus faecium* with the capacity to endure in media containing sodium chloride (NaCl) levels more than 22%, makes them well suited for performing fermentations of salted seafood and other highly saline products [83]. By comparison, plant fermentations tend to be less saline, and the prevailing LAB species that occur in these habitats are *L. fermentum* and *L. plantarum*, both of which can grow in media containing less than 6% NaCl [83].

In their study, Senthong et al. [82] isolated 306 LAB strains from samples of *Poo-Khem*, a traditional salted crab sold in Thailand. The isolates were screened for probiotic characteristics. They found that four strains including one strain of *Enterococcus thailandensis*, one strain of *L. plantarum*, and two strains of *L. fermentum* displayed notable probiotic traits such as tolerance to acid and bile salts, antimicrobial properties against common foodborne pathogens, and high cell surface hydrophobicity (an indicator of good adhesion ability to the human gastrointestinal tract). These probiotic traits validate their use as potential candidate LAB in starter cultures for controlled fermentation of seafood products like *Poo-Khem*. A similar study by Siripornadulsil et al. [84] found that strains of *Pediococcus pentosaceous* were the most isolated LAB from various traditional Thai fermented foods containing fish and pork. The strains exhibited probiotic properties such as tolerance to acidic conditions at pH 2, 0.3–0.5% bile salt and 1–14% NaCl. They also inhibited the growth of some pathogenic bacteria, including *Vibrio cholera*, *E. coli*, *Pseudomonas aeruginosa*, *Salmonella typhimurium*, *Bacillus cereus* and *Staphylococcus epidermidis*. Bacha et al. [85] evaluated the probiotic potential of LAB strains isolated from *Wakalim*, a traditional Ethiopian fermented beef sausage. Out of the 99 LAB strains that were evaluated, 44 tolerated pH 3.0 and bile salt concentration ≥ 0.3% for 3 h. The highest tolerance to pH 3.0 was observed among the pediococci (81.6%, 31/38) followed by lactobacilli (14.3%, 8/56), demonstrating their potential to enhance health when consumed through fermented sausages.

In India, Agaliya and Jeevaratnam [86] screened eight *L. plantarum* strains isolated from fermented idli batter for probiotic potential using in vitro assays such as bile tolerance, acid tolerance, transit tolerance in the upper human gastrointestinal tract, auto-aggregation, co-aggregation, hydrophobicity, susceptibility to various antibiotics, bile salt hydrolase assay, cholesterol assimilation and hemolysis. They found that the isolates could withstand pH values of between 2.5 and 8.5 as well as up to 0.3% bile for 4–6 h. Additionally, the isolates were able to withstand intestinal and stomach fluid expansion. Across all isolates, the auto-aggregation of the various strains of *L. plantarum* varied between 65 and 80% with co-aggregation ranging from 51 to 64% in pathogens like *Listeria monocytogenes* (MTCC 657) and *Escherichia coli* (MTCC 728). The isolates possessed â-galactosidase activity exhibiting 322 to 1000 milliunits of enzyme activity, and showed no hemolysis activity, indicating their probiotic potential which would attribute beneficial health effect to mankind [86]. Furthermore, Oluwajoba et al. [87] isolated probiotic LAB from Kunu-zaki, a traditional fermented beverage from Nigeria prepared from non-germinated sorghum and millet cereal grains. These LAB species demonstrated antibacterial activity against the reference strains of *Staphylococcus aureus*, *Escherichia coli*, *Pseudomonas aeruginosa*, and *Enterococcus faecalis*, as well as resistance to pH 3 and 3% bile. These species were mostly *Lactobacillus* species, and were identified *Pediococcus*, *Lactobacillus*, and *Lactococcus* species.

### 3.2. Emerging Probiotics from Dairy Fermented Foods

Dairy fermented foods have long been considered as the main source of probiotic products available in the market. Traditional dairy fermented products, such as yogurt, kefir, buttermilk, cheese, and cultured milk drinks, harbor a rich diversity of microorganisms, some of which have been found to possess exceptional health-enhancing properties beyond overall nutrition [5]. With the rise in the demand for functional foods, scientific interest in emerging probiotic strains from dairy fermented foods with improved survival in the gastrointestinal tract, enhanced adhesion, and distinct health benefits such as lactose digestion and immune modulation have also significantly increased [88]. In their study, Sharifi et al. [89] isolated 47 LAB from Iranian traditional yogurts, out of which 12 were considered potential probiotic candidates based on their ability to tolerate acidic pH and resistance to bile salts. Of these, six probiotic isolates belonged to *Pediococcus acidilacticii* and other six isolates to *Lactobacillus plantarum*, *L. brevis*, *L. fermentum* and *L. kefiri*. These organisms have been shown to occur naturally in cow milk [90]. Similarly, Hoque et al. [91] isolated *Lactobacillus* spp. from two regional yogurts in Bangladesh which demonstrated probiotic properties such as resistance to inhibitory substances like phenol (0.4%), NaCl (1–9%) and bile acid (0.05–0.3%). Additionally, the isolates demonstrated good growths in the presence of 1% NaCl and 0.3% bile acid.

Furthermore, Kefir, a traditional fermented dairy beverage made from the fermentation of different types of milk and kefir grains, is considered a healthy product with high nutritional value and has been associated with various health benefits [92]. This has spurred research towards the identification of potential novel probiotic strains present in kefir. Different studies have isolated and characterized microorganisms from kefir grains all over the world, some of them exhibiting probiotic properties. These include various fungi species such as 34 yeast strains isolated from 4 milky and 3 sugary kefir grains by Diosma et al. [93] which were identified as *Saccharomyces cerevisiae* (15 strains), *Saccharomyces unisporus* (6 strains), *Issatchenkia occidentalis* (4 strains), and *Kluyveromyces marxianus* (9 strains). In this research, *K. marxianus* CIDCA 8154 and *S. cerevisiae* CIDCA 8112 strains were selected and further studied. Both strains demonstrated the capacity to adhere to epithelial intestine-derived cells in vitro and to survive passage through the gastrointestinal tract of BALB/c mice. In another study, Chen et al. [71] isolated *Lactobacillus kefiranofaciens* M1 from Taiwanese milk kefir grain and investigated its effects on intestinal epithelial cells in vitro and on dextran sodium sulfate (DSS)-induced colitis in vivo. In vitro results showed that *L. kefiranofaciens* M1 could strengthen the epithelial barrier function by boosting transepithelial electrical resistance (TEER) and significantly increasing the level of the chemokine CCL-20, which plays a major role in immune signaling. In vivo, *L. kefiranofaciens* M1 reduced the severity of chemically induced colitis as shown by a significant attenuation of the bleeding score and colon length shortening. Additionally, it helped in balancing the immune response by decreasing the production of proinflammatory cytokines and increasing the production of the anti-inflammatory cytokine IL-10 demonstrating its potential to be used in fermented dairy products as an alternative therapy for intestinal disorders [71].

### 3.3. Emerging Probiotics from Other Unconventional Sources

Besides dairy and non-dairy fermented foods, other sources such as marine environments (e.g., seaweeds, fish gut microbiota, and marine sediments), fruit, flowers, and raw vegetables have also been explored as niches for the isolation of potential probiotic microbes for biotechnological application or functionalization of foods [21,32]. These include some flowers which are colonized by fructophilic bacteria (that is, a preference for D-fructose over D-glucose) belonging to the genus *Fructobacillus* [94]. These bacteria produce mannitol and acetate rather than ethanol which is useful for biotechnological applications considering that mannitol is a low-calorie polyol frequently used in the food sector [95]. The potential of these as probiotics have been further evaluated by recent studies like that of Patil et al. [29] that isolated strains with fructophilic characteristics such as *F. fructosus* MCC 3996 from the nectar of *Butea monosperma* flower. The strain was evaluated in vitro for probiotic characteristics and it was found to demonstrate resistance to acidic environments and gastric juice, improved auto-aggregation even when hydrolytic enzymes were present, hydrophobicity, co-aggregation with pathogenic bacteria, and the absence of hemolytic activity [29]. Similarly, another study conducted in China by Sakandar et al. [96] isolated fructophilic LAB (FLAB) from various flowers and fruits and evaluated them for probiotic characteristics. The isolated strains include *F. pseudoficulneus* JNGBKS1 and JNGBKS3 from peach and banana, respectively; *F. fructosus* JNGBKS2 and JNGBKS4 from narcissus and sunflower, respectively; *F. durionis* JNGBKS5 from kiwi fruit; *L. kunkeei* JNGBKS6, JNGBKS7, and JNGBKS8 from narcissus, yellow rose, and pink rose, respectively. In comparison to the other FLAB strains that were isolated, *L. kunkeei* strains demonstrated the maximum capacity to tolerate various low pH levels and bile acid concentrations together with maximal effects on antipathogenic activity, cholesterol assimilation, and hydrophobicity [96].

Similarly, *Weissella paramesenteroides* which has gained considerable attention in recent years as bacteriocin and EPS producers, was isolated in the study by Pabari et al. [43] from different fruits (cherry, sapota, banana, orange, and plum). In this study, five *W. paramesenteroides* strains, namely, FX1, FX2, FX5, FX9, and FX12, were isolated and screened for probiotic and prebiotic traits. Two strains FX5 and FX9 were chosen for prebiotic utilization investigations using thin layer chromatography (TLC) and high-performance liquid chromatography for the synthesis of short-chain fatty acids (SCFAs) based on their functionality. Since only the top low molecular weight fractions vanished from cell-free supernatants (CFS), the TLC profile demonstrated that these two strains were able to use low molecular weight fructooligosaccharides (FOS) and galactooligosaccharides (GOS). GOS utilization ability was demonstrated by increased β-galactosidase activity which is related to galactose buildup in the residual CFS of GOS. Both strains demonstrated antimicrobial activity against *S. aureus* and *E. coli*, as well as significant SCFA synthesis when prebiotic was present, indicating their potential as synbiotics [43].

From marine ecosystems, the exploration of probiotic LAB has also been reported. In their study, Das et al. [32] evaluated the probiotic potentials of three marine LAB isolates characterized as *L. casei* SB71, SB73 and SB93. They found that these isolates demonstrated high level of gastrointestinal survival, marked cholesterol assimilation, adherence to Caco-2 cells, and inability to form biogenic amines. The isolates were also tolerant to NaCl, bile and low pH. Notably, all three isolates produced bacteriocins that exhibited sustained antimicrobial activity against *V. cholerae*, whereas the EPS from the SB93 strain notably disrupted the adherence of bacteria and promoted remediation of cadmium and lead [32].

## 4. Application of Probiotic Strains in Functional Food Development

Functional application of probiotic strains depends not only on their benefits to health but also on their ease of application in real food products. As previously discussed in Section 2, the biological and safety characteristics of each strain, including stress tolerance, adhesion capacity, antipathogenic activity, and confirmed safety are critical determinants of its practical performance in food systems. In functional food design, these properties must align with formulation requirements, processing stability, and sensory quality to ensure that probiotics remain viable and efficacious throughout the shelf life of the product [2,10]. Hence, the development of probiotic-enriched foods therefore focuses on major aspects such as the compatibility of the strain with the food matrix, technological strategies such as encapsulation to enhance survival and stability, and innovative product formulation to maintain probiotic delivery and consumer acceptability. Together, these factors determine whether a probiotic product will deliver the assured health benefits to consumers.

### 4.1. Food Matrix Compatibility

Determining the compatibility of the probiotic strain with the selected food matrix is very crucial when developing functional foods. This is an important consideration which has encouraged the search for newer probiotic strains, amongst other reasons. The addition of traditional probiotic strains of dairy origin into plant-based foods typically involves significant hurdles. Most commercial probiotic strains fail to maintain adequate survival rates in non-dairy substrates because these environments can be harsher due to factors like reduced pH, different levels of nutrients, or the presence of antimicrobial phytochemicals [21]. The ability of a strain to maintain metabolic activity and cell viability during food processing and storage depends on these parameters, which can vary significantly across food types. Fermented fruit juices or cereal-based beverages for example, often present acidic conditions and oxygen exposure that can reduce the stability of probiotics. However, matrices rich in fibers, sugars, or polysaccharides (such as β-glucans or inulin) can act as protective agents, enhancing the survival of the microorganisms during storage and gastrointestinal transit [97]. By comparison, LAB found naturally in plant matrices tend to be more likely to survive and maintain life in such environments, increasing their probability to deliver probiotic benefit when applied to vegetable, fruit, or cereal-food products [98]. The use of plant-derived LAB probiotics can help to broaden the range of functional foods which can be fortified with probiotics to meet the demands of both traditional and emerging consumer markets.

Another key determinant for matrix compatibility is the influence of probiotic addition on sensory and textural properties. The interaction between the matrix and probiotic cells can impact flavor, aroma, viscosity, and appearance, which ultimately determines consumer acceptability. Studies have shown that supplementation of *L. plantarum*, *L. fermentum*, and *L. brevis* in non-dairy matrices such as oat, soy, and fruit-based beverages maintained viable counts above 10^7^ CFU/mL under storage conditions preserved acceptable sensory characteristics [99]. Microencapsulation techniques, prebiotic enrichment, and co-fermentation using compatible starter cultures have also been explored further to maintain probiotic stability and incorporation into various matrices [100]. These approaches balance microbial performance with technological viability and customer acceptability, and these are critical for effective product development and marketing.

### 4.2. Encapsulation of Probiotics to Improve Shelf-Life and Viability

As indicated by the World Gastroenterology Organization, the effective dose of probiotics necessary to deliver health benefits differs widely with the microbial strain and type of product [101]. Although many commercial probiotic foods or supplements are typically in the 1 to 10 billion CFU (colony-forming units) per dose range, there are strains that are inoculated at lesser doses, and others that require higher concentrated doses to have an appreciable effect [100]. It is generally recommended that a probiotic food product has a minimum of 10^6^ to 10^9^ CFU per gram and daily intake equals at least 100 g or milliliters to have an effective therapeutic effect [102]. However, ensuring that live probiotic cells do, in fact, reach the consumer in efficacious numbers is challenging. During food storage and processing, probiotic organisms encounter several stressors such as heat, moisture, light, oxygen, osmotic stress, and exposure to high acidity and digestive enzymes when consumed [103]. In the dairy industry, for instance, procedures such as pasteurization, homogenization, agitation, freezing, drying, and even the addition of salt or other antimicrobial compounds can drastically reduce the viability of probiotic bacteria [104]. Studies have shown that to retain a shelf-life of up to 12 months at room temperature, the water activity of a probiotic product must be extremely low, i.e., preferably less than 0.25, to prevent microbial loss [105].

In view of these challenges, the initial cell count, strain selection, formulation pH, processing steps, and the food matrix itself are all significant parameters that dictate if probiotics survive and remain effective [106]. To overcome these limitations, advanced encapsulation technologies have been an effective approach in probiotic food design. Techniques like microencapsulation, which involves using methods like spray drying, freeze-drying, or nano-coating, gives a protective layer to sensitive probiotic cells [105]. The protective layer cushions the microbes against the stressful processing conditions and the acidic environment of the stomach and small intestine so that more viable cells can pass through to the gut to exert their effects [104]. Additionally, encapsulation may be employed to conceal any unfavorable taste or odor of live cultures and deliver a long shelf-life without compromising sensory qualities [106]. Importantly, selecting the right encapsulation material is essential to the success of these technologies. In addition to being safe for human consumption and cost-effective, the material should ensure that the probiotic cells are protected throughout food processing and storage, and during delivery in the gastrointestinal tract [107]. Materials with gel-like structures and greater dry matter content tend to be more effective since they create a stable matrix for the living cells. Various natural polymers and hydrocolloids including alginate, xanthan gum, chitosan, carrageenan, cellulose, and certain fats and proteins are often utilized in formulating microcapsules, each bearing specific characteristics that can increase encapsulation effectiveness and probiotic viability [108]. Hence, pairing encapsulation technologies with the careful screening of robust emerging strains would allow food companies to increase the bioavailability and stability of probiotics in dairy and non-dairy functional foods, widening product possibilities and consumer trust in probiotic health claims.

Studies have reported on the encapsulation of bacteriocins from LAB for effective activity against spoilage and pathogenic organisms, including spores [109,110]. However, recent studies have reported the use of encapsulated probiotic cells themselves in the production of functional foods to mitigate the challenges faced by free probiotic cells in high acidic environmental conditions. For instance, Afzaal et al. [111] evaluated the effect of microencapsulation on the viability and stability of probiotic bacteria in yogurt and simulated gastrointestinal conditions. They incorporated free probiotic (*L. acidophilus*) into the yogurt during its preparation and found that the survival of the bacteria decreased from 9.97 log CFU/mL on first day to 6.12 log CFU/mL on day 28. The free probiotic cells also showed very poor survival during in vitro gastrointestinal assay. To improve the viability of probiotics, they used the extrusion technique to encapsulate the bacteria using sodium alginate and carrageenan. They found that the encapsulation improved the viability of the probiotics in the prepared yogurt and gastrointestinal tract. At zero-day, the probiotics encapsulated with sodium alginate and carrageenan had cell counts of 9.91 and 9.89 logs CFU/mL, respectively, which dropped to 8.74 and 8.39 log CFU/mL by the end of the day. The study also demonstrated that sodium alginate was a better encapsulating material for probiotics in comparison to carrageenan. [111]. A similar study by Chen et al. [112] evaluated the effect of xanthan-chitosan-xanthan (XCX) encapsulation system on the viability of *Bifidobacterium bifidum* BB01 in yogurt during 21 days storage at 4 °C and 25 °C, respectively. They used chitosan as an inner layer to coat the xanthan-probiotic (XC) and xanthan was again used as an outer layer for coating chitosan-xanthan-probiotic particles. The results showed that the *B. bifidum* BB01 encapsulated with these multilayer hydrogels remained viable for up to 21 days in yogurt stored at both refrigeration and room temperatures, with significantly (*p* < 0.05) better survival than free, unencapsulated cells. In comparison to the free cells, all the microcapsules showed higher cell survival of probiotic in the simulated gastric fluid and bile salt solution. Notably, the XC microcapsules exhibited better release profile than XCX microcapsules in the simulated intestinal fluid. These findings demonstrate how carefully designed biopolymer encapsulation can maintain probiotic viability during storage and release them in the gut, facilitating the development of robust functional foods.

In non-dairy functional foods like fruit juices, the use of encapsulated probiotics has also been reported. Dias et al. [113] produced a powdered probiotic passion fruit juice through microencapsulation of *Bifidobacterium animalis* subsp. *lactis* BB-12 with maltodextrin and/or inulin as encapsulating agents. Powders were tested for physical, thermal, and microbial stability for 30 days of storage at 25 °C and 4 °C. Their results showed that inulin, either alone or with maltodextrin, was a better protectant for maintaining the probiotic viability, especially at 25 °C, compared with maltodextrin alone. All the powders maintained flow characteristics and solubility, though differences in carrier materials influenced moisture content, particle shape, and color over time stability. Overall, the study demonstrated that probiotic encapsulants combined with controlled storage conditions may be utilized to promote the shelf life of probiotics in non-dairy food systems like fruit juices and enable the development of stable, functional probiotic drinks. In another study, internal gelation was used to encapsulate probiotic bacteria, including *L. acidophilus* and *B. bifidum*, in alginate beads with a mean diameter of 54.25 ± 0.18 µm. The pasteurized grape juice was then mixed with both encapsulated cells and free cells as control samples, and the mixture was kept for 60 days. The bacteria in the encapsulated samples had significantly (*p* < 0.05) higher survivability at the end of the storage period than the sample with free bacteria cells (8.67 ± 0.12 and 7.57 ± 0.08 log CFU/mL for *L. acidophilus* and 8.27 ± 0.05 and 7.53 ± 0.07 log CFU/mL for *B. bifidum* for the encapsulated and free forms, respectively) [114].

### 4.3. Role of Artificial Intelligence in the Discovery, Characterization, and Application of New Probiotics

Artificial intelligence (AI) and machine learning (ML) methods are becoming increasingly applied across the probiotic pipeline, from rapid genome and metagenome screening to prediction of colonization outcome, risk assessment of safety, formulation and delivery optimization. These technologies support traditional wet-lab screening by (i) discovering candidate strains with desirable genetic and functional properties, (ii) automating detection of risk factors (e.g., antimicrobial resistance genes, mobile elements), and (iii) modeling host–microbe and community-level responses for improved strain selection and formulation [115,116]. For example, the study by Sadeghi et al. [117] demonstrated the use of ML models for the selection and prioritization of 15 out of the 144 LAB strains screened in their study, based on their combined potential probiotic properties and antimicrobial activity for application in plant tissue cultures. This study highlights the ability of AI-driven models to enhance the precision and efficiency of probiotic screening and selection processes. Another study by Zhang et al. [118] analyzed 31,977 LAB genomes and identified over 130,000 secondary metabolite biosynthetic gene clusters (BGCs) using machine learning models to predict their antimicrobial potential. Through the integration of metagenomic and metatranscriptomic analyses, the researchers linked LAB-derived metabolites to protective and regulatory functions in the human microbiome, demonstrating how AI and omics integration can accelerate the discovery and functional characterization of novel probiotic metabolites with therapeutic potential.

Bacteriocin/RiPP mining software such as BAGEL (and BAGEL4) are still needed to identify putative antimicrobial peptides encoded in genomes and are used routinely in combination with ML pipelines for functional annotation [119]. AI classifiers improve the detection of antibiotic resistance genes (ARGs) and mobile genetic elements in high-throughput sequencing data. For instance, DeepARG, a deep-learning program trained on hand-curated ARG databases, has been shown to provide sensitive and accurate ARG annotation from both isolate and metagenome-derived genomes, a crucial step in ruling out probiotic candidates that may be responsible for horizontal transfer of resistance [120]. Similarly, ML tools like PlasFlow, PlasClass and Deeplasmid help identify plasmid-derived contigs and differentiate them from chromosomal sequences, which is important in assessing the mobility risk of identified resistance or virulence genes [121]. Furthermore, newer specialized tools such as metaProbiotics has incorporated language-model to directly mine metagenomes rapidly for probiotic-like genome bins, facilitating recovery and priority of candidate strains from large data sets and performing better on test benchmarks [122]. ML can also be used in the prediction of functional outputs such as short-chain fatty acid production or other metabolites from strain mixtures and environmental conditions, allowing in silico optimization of strain consortia prior to laboratory validation. For example, Westfall et al. [116] developed an artificial model of the human gastrointestinal tract called ABIOME (A Bioreactor Imitation of the Microbiota Environment), for the prediction of metabolic activity of probiotic formulations and hence therapeutic potential with machine learning tools. The combination of ABIOME and the multivariate adaptive regression splines (MARS) model led to the identification of several probiotic combinations that stimulated synergistic production of bioavailable metabolites, each with a different therapeutic capacity. Collectively, these examples demonstrate how AI and ML approaches significantly enhance the precision, scalability, and predictive power of probiotic discovery and formulation, bridging computational insights with practical applications in food and health. However, this is not without limitations. While the integration of AI and ML has led to the acceleration of probiotic candidate selection and hypothesis generation, outputs should be interpreted cautiously. ML predictions are strongly dependent on training data quality and representativeness; models trained from sparse or biased training data may generate false positives/negatives [123]. Experimental validation (in vitro, in vivo, and human trials) is still required to confirm predicted safety, functionality, and efficacy [2]. Furthermore, regulatory acceptance of AI evidence is more in its infancy; open publication of model structures, training data, and performance metrics will be crucial to industry and regulator uptake. Finally, the integration of clinical and individual microbiome data creates data-privacy and ethics problems that must be addressed before the wide implementation of personalized probiotic products [124].

## 5. Health Benefits and Mechanisms of Action of Probiotics

The beneficial effects of probiotics on the human body are usually because of one or a combination of several processes, including altering the intestinal microbiota, blocking pathogen attachment sites, production of metabolites, modifying the host immune response, and nutrient competition [21].

### 5.1. Immunomodulatory and Anti-Inflammatory Effects

Probiotics play a crucial role in enhancing the host’s immune defenses by influencing how the body recognizes and responds to invading pathogens in the host. Studies have shown that probiotics can navigate through the intestinal mucus layer, in which they survive, interact with the gut epithelial cells and promote the modulation of innate and adaptive immune processes. In their study, Song et al. [125] found that *Lactobacillus brevis* B13-2 strain isolated from kimchi exhibited immunomodulatory activity by inducing the expression of some cytokines (IL-1β, TNF α, and IL-6) and iNOS. Interestingly, the observed immunomodulatory effect was in addition to its probiotic effects such as tolerance against artificial gastric acid and bile salts, adherence to HT-29 cells, and absence of β-glucuronidase production. In an in vivo experiment, male Swiss albino mice that consumed 10 CFU/mL of fruit-derived *S. cerevisiae* IFST 062013 showed increased humoral and cell-mediated immunity, and a stimulation of T-lymphocyte-specific proliferative response. Additionally, the probiotic improved host immunity by inducing pro- and anti-inflammatory mediators and preserving the equilibrium of Th1 and Th2 cytokines [126]. In another study by Ornellas et al. [127], it was demonstrated that mice that were given a single dosage of *L. plantarum* 81 and *L. plantarum* 90 isolated from cupuaçu (*Theobroma grandiflorum*) fermentation (10^8^ CFU/mL) produced more of the regulatory cytokine IL-10 and less of the proinflammatory cytokines IFN-γ and IL-6, leading to an exhibition of anti-inflammatory effect.

### 5.2. Effect on Cardiovascular Health

There is growing evidence of the role of probiotics in cardiovascular health maintenance, particularly their cholesterol-lowering property. Certain probiotic strains have been found to help decrease serum cholesterol levels, thus lowering heart disease risk [128]. One of the major ways probiotics achieve this is by decreasing the solubility of cholesterol in the intestine, which decreases the quantity absorbed into the blood [50]. In addition, many probiotics produce bile salt hydrolase (BSH) enzymes that breaks/deconjugate the bond between a conjugated bile acid and its attached amino acid (glycine or taurine), releasing the bile acid in its free (deconjugated) form. Such deconjugated bile salts are readily removed from the body through feces. Additionally, since cholesterol is a major precursor of bile acid synthesis, this increased removal of bile acids provokes the body to convert more circulating cholesterol into new bile salts, resulting in lower total serum cholesterol levels [129]. The in vivo study by Cavalcante et al. [130] demonstrated that male Wistar rats fed with high-fat diets treated with fruit-derived *L. fermentum* 296 had improved biochemical and cardiovascular parameters that are altered in cardiometabolic disorders. Similarly, another in vivo study by Costabile et al. [131] evaluated the cholesterol reducing capacity of *Lactobacillus plantarum* ECGC 13110402 in 49 adults with normal to mild hypercholesterolemia. The study was run in a parallel, double blind, placebo controlled, randomized design in which the active group of volunteers ingested 2 × 10^9^ CFU encapsulated *Lactobacillus plantarum* ECGC 13110402 twice daily for a period of 6–12 weeks. Results showed that this probiotic strain reduced LDL cholesterol by 13.9% among the subjects who had relatively higher initial cholesterol levels and reduced total cholesterol by as high as 37.6% among subjects with higher initial levels. Also, participants over 60 years experienced a significant decrease in triglycerides (53.9%) and a rise in healthy HDL cholesterol (14.7%). Importantly, lowering of systolic blood pressure (6.6%) was also observed in the trial, and the probiotic proved to be tolerant without any gastrointestinal side effects. This demonstrates that probiotic strains like *L. plantarum* ECGC 13110402 hold promise in having a facilitative role to play in cholesterol management and reduction in cardiovascular risk, either as add-on or standalone in place of conventional therapy.

### 5.3. Anti-Anxiety and Anti-Depression

There is increasing evidence that symptoms of depression and anxiety can be eased by certain probiotic strains. This hypothesis was first evidenced in the 1970s by Tannock and Savage [132], who reported that stress in mice markedly reduced their levels of gut *Lactobacilli*, implying a connection between mental status and gut microbiome. Since then, the gut–brain axis has been a key focus of psychobiotic research. Studies have shown that probiotics may exert a positive effect on mood and mental health via numerous mechanisms, such as the enhancement of immune response and extension of production or availability of mood-regulating neurotransmitters like serotonin [133]. Animal models, for instance, rats in forced swim tests, have been used for the preliminary screening of probiotic strains for antidepressant-like effects. These experiments also measure changes in behavior alongside biochemical markers like neurotransmitter levels of serotonin, dopamine, and noradrenaline, or their precursors like tryptophan [50].

In humans, clinical trials have demonstrated that probiotic supplementation reduces symptoms of depression, anxiety, and stress as assessed using conventional scales like the Beck Depression Inventory (BDI), the Beck Anxiety Inventory (BAI), or the Hamilton Anxiety Rating Scale (HAM-A). Reductions in the levels of stress hormones like cortisol and adrenocorticotropic hormone have also been reported in probiotic-treated groups compared to controls, indicating a modulating effect on the body’s stress response [50,134,135]. In their study, Akkasheh et al. [136] evaluated the impact of probiotic supplementation on metabolic profiles, serum high-sensitivity C-reactive protein (hs-CRP), indicators of oxidative stress, and depression symptoms in patients with major depressive disorder (MDD). The study was run in a randomized, double-blind, placebo-controlled design in which patients were randomly allocated into two groups to receive either probiotic supplements (*n* = 20) or placebo (*n* = 20) for 8 weeks. The probiotic capsule consisted of three viable and freeze-dried strains: *Lactobacillus acidophilus* (2 × 10^9^ CFU/g), *Lactobacillus casei* (2 × 10^9^ CFU/g), and *Bifidobacterium bifidum* (2 × 10^9^ CFU/g). Results showed that the BDI scores of patients who received the probiotic supplements were considerably lower than those of the placebo group (−5.7 ± 6.4 vs. −1.5 ± 4.8, *p* = 0.001). Furthermore, when compared to the placebo, there were notable drops in serum hs-CRP concentrations (−1138.7 ± 2274.9 vs. 188.4 ± 1455.5 ng/mL, *p* = 0.03), insulin resistance as determined by the homeostasis model (−0.6 ± 1.2 vs. 0.6 ± 2.1, *p* = 0.03), and serum insulin levels (−2.3 ± 4.1 vs. 2.6 ± 9.3 μIU/mL, *p* = 0.03). This highlights the potential of probiotics to serve as an accessible, complementary approach for supporting mental health, especially when used in combination with conventional therapies. However, more robust, large-scale human studies are still needed to confirm which specific strains and dosages provide the most consistent benefits for mental well-being.

### 5.4. Antimicrobial Activity and Modulation of Gut Microbiota

One of the most important functional properties of probiotics is their ability to produce antimicrobial compounds, including organic acids, short-chain fatty acids (SCFAs), and bacteriocins that help to create an unfavorable environment for pathogenic microorganisms [62]. Nisin, for instance, which is produced by *L. lactis*, has been reported to inactivate growth of vegetative spore-forming Gram-positive bacteria by binding to lipid II, disrupting cell wall biosynthesis and facilitating the formation of pores. This antimicrobial action has also been reported in emerging probiotics. For example, Mabeku et al. [137] demonstrated in their study that LAB strains isolated from fermented *Theobroma cacao* fruit juice successfully inhibited the bacterium *Helicobacter pylori*, which is implicated in gastritis, gastric ulcer, and even certain types of stomach cancer. The authors attributed the antimicrobial activity of the LAB strains to their ability to produce bacteriocins that led to the inhibition of the pathogen’s growth. This antimicrobial activity is not only a welcome alternative to traditional antibiotics, but a necessity in the extension of the shelf life and safety of foods.

In addition to their direct inhibition of pathogenic microbes, probiotics are also significantly crucial in balancing gut microbiota. A healthy colon is dominated by beneficial anaerobic bacteria. However, when dysbiosis (microbial imbalance) occurs, undesirable microbes such as hemolytic *Staphylococcus* species have the potential to overgrow and induce inflammation or impair gut barrier function [50]. Studies have shown that probiotics in food supplements play crucial roles in reestablishing gut microbiota balance by stimulating growth of beneficial anaerobes while suppressing the proliferation of unwanted bacteria. For instance, yeasts like *Wickerhamomyces subpelliculosus* and *Pichia kudriavzevii* used as a starter culture in a cornelian cherry beverage demonstrated in vitro to be effective at improving the numbers of beneficial anaerobic bacteria and inhibiting *Staphylococcus* spp. growth, even better than noted prebiotics like fructooligosaccharides [138]. However, more stringent, heterogeneous human trials are needed to establish comprehensively the scope and uniformity of the antimicrobial and microbiota-modifying activity of emerging probiotics.

## 6. Translational Insights: Market Trends and Consumer Acceptance of Probiotics in Functional Foods

The technological and biological attributes of probiotics strains that enable them to withstand food processing, storage, and gastrointestinal transit also enhance their commercial potential/viability. This becomes more relevant as scientific understanding in this field grows and is utilized to elucidate mechanisms of stress resistance, adhesion, and safety. These insights increasingly inform the strategic development and positioning of novel functional food products, fast-tracking modern probiotics from laboratory characterization to commercial product development. This is further shaped by the evolving consumer preferences, regulatory frameworks, and innovations in food formulation.

As highlighted, functional foods fortified with probiotics have moved from a niche category centered on fermented dairy to a diversified global marketplace spanning beverages, snacks, infant and medical nutrition, and shelf-stable categories. This has increased the market reach of these products. Furthermore, with the growing scientific interest in gut microbiome research, combined with advances in formulation and quality control, product formats which can be fortified with live microorganisms have broadened, together with delivery methods and a rapid emergence of personalized, microbiome-based products. However, there are still some concerns on how scientific validation can be standardized and streamlining of regulatory guardrails to shape consumer acceptance.

### 6.1. Market Growth of Functional Foods and Probiotics

Over the years, the global probiotic market has grown significantly. Industry analyses estimate the global probiotics market to be on the order of tens of billions USD today, with reported valuations ranging from ~USD 70–90 billion in the mid-2020s and projected growth to >USD 100 billion within the next five to ten years depending on scope, with one estimate placing the value at USD 87.70 billion as of 2023, with a projected annual growth rate up to 14% in 2030 (USD 220 billion) [139]. This growth is driven by the converging consumer demands for diverse classes of probiotic-fortified functional foods, a shifting interest in digestive comfort, immune support, and holistic wellness through better-for-you snacking and beverages; and the proliferation of plant-based matrices that invite fermentation and fortification [140]. Conventional strains of probiotics including *Lactobacillus* and *Bifidobacterium* species are still utilized in the bulk of food applications because their safety is well-documented, manufacturing is scalable, and regulatory pathways are comparatively clear [106]. These strains dominate categories where cold-chain distribution is practical and consumer familiarity is high, especially yogurt, kefir, fermented milks, and cultured plant-based alternatives. In parallel, with encapsulation technologies and the adoption of heat- and acid-tolerant spore formers such as *Bacillus coagulans*, more conventional foods can be fortified with these emerging probiotics, when properly characterized at the strain level and manufactured to avoid toxigenicity [24]. This allows for the use of probiotics in ambient beverages, nutrition bars, baked goods, confectionery, and cereals, which are food forms that were historically resistant to live-culture inclusion. The growth in the probiotic market varies with regions, with the Asia-Pacific region being the fastest-growing and often largest regional market for probiotics and probiotic-fortified foods, driven by large population bases, rising disposable income, and cultural acceptance of fermented products [141].

### 6.2. Consumer Awareness and Preferences

Consumer awareness has shifted from generic “good bacteria” messaging to more nuanced expectations about strain identity, dose, and evidence. Many consumers now look for labeled genus-species-strain designations, guaranteed CFUs through end of shelf life, and clear benefits linked to everyday outcomes such as regularity, tolerance of lactose and Fermentable Oligo-, Di-, Mono-saccharides and Polyols (FODMAPs), reduced bloating, or immune support during seasonal challenges [142,143]. Trust is built through strategies that allow consumers to make informed choices. This is promoted through third-party certifications of good manufacturing practices, and QR-code transparency that links to certificates of analysis and study summaries [144]. Sensory performance remains critical: despite health motivations, products must deliver desirable taste, texture, and aroma. Fermented dairy continues to set the benchmark for probiotic palatability, but growth in plant-based matrices has diversified preferences toward oat, coconut, and soy substrates, where fermentation can mitigate off-notes, reduce sugars, or improve protein digestibility, provided starter cultures and probiotics are harmonized to avoid sensory defects. Furthermore, cultural factors also shape consumer perception and acceptance of fortified foods: in East Asian markets, traditional fermented foods and beverages create a receptive baseline, whereas in parts of Europe regulatory conservatism raises the bar for claims but fosters trust in products that pass it [145]. Across geographies, clean-label expectations (short ingredient lists, minimal processing, non-GMO where relevant) and sustainability credentials (low-impact packaging, responsible sourcing of prebiotics) increasingly influence purchase decisions [146].

One of the key determinants of the success of functional foods with probiotics, particularly with emerging strains recently, is consumer education. It has been identified that early adopters of probiotic and other health-related food products are younger, health-conscious consumers who are extremely trend-sensitive as a result of what they observe on social media and who place an extremely high value on the use of naturalness in foods [147,148,149]. Alongside this, consumer attitudes towards food, health, and nutrition have become more complex and fragmented [1]. Part of the reason is internet connectivity that has empowered people to instantly search for information on food products, ingredients, and health claims on their phone. This easy access to (potentially contradictory) information has emboldened consumers to be more skeptical of expert advice, especially younger consumers who can be skeptical of traditional health professionals [150]. Importantly, many consumers confuse probiotics, prebiotics, postbiotics, and fermented foods; others assume any fermentation confers probiotic effects, which is not the case unless specific strains and doses are delivered alive where benefits occur. There is need to provide plain-language explanations, distinguishing live probiotic strains from general starter cultures, clarifying that benefits are strain specific, and explaining why CFUs must be guaranteed through the end of shelf life. Equally, responsible communication includes reminders that probiotics complement, not replace, medical care for diagnosed conditions. For example, variations in manufacturing processes may influence the ratio of live to dead bacteria in a probiotic product. It is certain that during manufacture, storage, or freeze-drying, some probiotic cells will die. To compensate and meet the given live cell count on the label, companies will often add extra live cells, a process called “overfilling”, in such a manner that the product will continue to deliver the minimum indicated CFU at the time of consumption [151]. However, current labeling conventions usually report the number of live, viable bacteria per dose as CFU but not the total number of bacterial cells consumed, including dead cells. Consumers are therefore sometimes unknowingly consuming more total microbial load than reported on the label. While dead cells might infrequently exert beneficial health effects [125], this lack of clear information can affect consumer confidence and raise concerns about transparency and informed choice. Hence, it has become important for clear, simple labeling and consumer education to be considered when introducing new probiotic strains or new microbial ingredients. This is because trust that product labeling is clear and accurate is another key determinant of the way consumers perceive emerging probiotic products, since consumers would like to know what strains were included, what specific health benefit they may provide, and if the statements are supported by trustworthy science. Stricter guidelines and improved communication are therefore needed to help them understand exactly what CFU means, how production methods influence viability, and the role of both live and dead cells. While companies and regulators face the challenge of balancing scientific accuracy with simple, understandable communication that builds trust, fulfilling this need for clarification will be key to winning more acceptance of new probiotic products in an increasingly skeptical but health-conscious market.

### 6.3. Personalized Nutrition and Microbiome-Based Products

The rapidly expanding catalogue of next-generation and engineered probiotic organisms amplifies personalization potential of functional foods. Candidate commensals such as *Akkermansia* and *Faecalibacterium* spp. are being explored for the amelioration of metabolic and inflammatory conditions, although most remain investigational for general food use [42]. Engineered LAB that secrete enzymes (for example, lactase for lactose maldigestion) or other bioactive peptides foreshadow a future of function-specific foods. Even within conventional species, personalization can occur by selecting strains with distinct bile-salt hydrolase activity, exopolysaccharide profiles that influence immune signaling, or carbohydrate utilization pathways matched to an individual’s habitual diet [152]. Synbiotic co-design takes this further by pairing a strain with the prebiotic it best metabolizes, improving engraftment and persistence. Personalization is reshaping the probiotic food landscape through three intersecting pathways: diagnostics, targeted strain selection, and adaptive delivery [153]. Consumer microbiome testing, typically 16S rRNA sequencing with increasing adoption of shotgun metagenomics has become a gateway to individualized recommendations. While methodological limitations remain, these tests provide relative abundance profiles, diversity metrics, and functional potential proxies that can be mapped to diet and probiotic suggestions. Of recent, it is now possible to translate such readouts into tailored food plans and curated assortments of probiotic-fortified products, sometimes combined with wearable-derived lifestyle data and short-term biomarkers (e.g., breath hydrogen or glycemic responses) [154]. A growing number of programs use iterative algorithms where consumers submit periodic samples and symptom diaries. These are utilized together with advanced technologies including blockchain and algorithms to update the strain mix, prebiotic pairing, and intake timing to nudge the gut microbiome toward desired functional states [155,156].

Despite promise, personalized probiotic foods face scientific and ethical hurdles. Inter-individual variability in microbiome ecology and host genetics complicates attribution: the same strain may have divergent effects depending on baseline community structure, diet, and transit time [157]. Also, many endpoints of interest such as mood, stress resilience, skin appearance are multidimensional and require careful study design to avoid over-interpreting placebo-responsive signals. Also, data privacy and consent frameworks must evolve to protect genomic and health-related information collected through testing programs. These are issues which need to be addressed as the market for personalized probiotic foods grows.

## 7. Challenges and Future Perspectives

While the global interest in probiotics has continued to grow over the years, driven by consumer demand for health-promoting foods, the use of novel and emerging microbial strains presents new challenges and raises questions about safety, harmonized regulatory frameworks, and scientific rigor.

### 7.1. Regulatory and Safety Considerations

As research continues the exploration of emerging microbial strains from different unconventional sources, it is important to ensure the safety of these products for human use. Regulatory frameworks like the Generally Recognized as Safe (GRAS) status in the United States and the Qualified Presumption of Safety (QPS) list in the European Union provide structured approaches for assessing the safety of microbial strains used in food and supplements. The QPS concept for instance, was developed by the EFSA as a generic risk assessment method for biological agents used in the production of food and feed to streamline and standardize the evaluation of notified biological agents across the EFSA’s numerous Scientific Panels and Units [36]. This technique makes it easier to evaluate and approve low-risk microorganisms with a pre-established history of safe use, while allowing for a more targeted use of the available resources for agents with higher risk potential [158]. If under the QPS system a strain can be unequivocally placed in a taxonomic group already present on the QPS list, then a very limited number of additional safety checks could be required. Such a system would rely on four broad pillars namely: correct taxonomic identification; sufficient knowledge and literature related to safe use; evidence ruling out pathogenic or virulent nature; and assurance of the end use and effectiveness of the microorganism in the final product [36,72,159].

In addition to QPS, the EU-funded PROSAFE project (Biosafety Assessment of Probiotic Lactic Acid Bacteria for Human Consumption) was launched to increase evidence-based recommendations to assess the safety of probiotics for use in human beings [72]. In 2006, PROSAFE specialists outlined operational measures to guide future probiotic development. These included calling for the application of proper molecular identification methods, such as ribosomal DNA sequencing by qualified laboratories, to ensure taxonomic LAB classification [160]. Strains should also be deposited in established public collections for reference, with access being controlled. One of the key suggestions was that strains with higher or unusual antibiotic resistance should not be put into the food chain for human or animal consumption without extensive risk evaluation [74]. Similarly, probiotic strains with documented possession of established virulence genes for example, some *Enterococcus* species, are to be avoided at all costs. It was also ascertained that fringe properties such as bile acid deconjugation or broad adhesion capability, were insufficient metrics to base probiotic safety decisions on independently [72]. Furthermore, PROSAFE outlines that the use of animals for safety was deemed inferior to human trials as in vivo safety tests of probiotic ability to adhere to human gut should preferably be evaluated by well-designed human clinical trials which ideally should be randomized, placebo-controlled and double-blind. However, if animal models are to be used, rat endocarditis model was suggested as being most informative because it allows for the assessment of potential pathogenicity and translocation of probiotic strains to sterile sites, such as cardiac tissue, thereby providing a sensitive and reproducible system to detect virulence traits under conditions closely simulating the pathophysiology of infective endocarditis in humans [161].

Globally, the 2002 FAO/WHO guidelines remain authoritative in laying down overall principles for safe food use of probiotics [46]. These expert guidelines coincide mostly with those of PROSAFE. They highlight the importance of having a documented history of safe use, absence of transferable antibiotic resistance and pathogenic virulence factors, and additional testing for some metabolic functions such as D-lactate production or toxin production in species known to produce toxins. They also recommend checking hemolytic activity if appropriate and observing potential side effects throughout human trials. Lastly, the guideline notes the necessity of continuing post-market surveillance to monitor any side effects when products reach consumers. However, while these guidelines emphasize key elements of safety, they lack full details on how to conduct these tests or interpret their results in practice.

In the United States, the regulation on probiotics remains fragmented as there is no official legal definition of “probiotic” at the federal level [72]. The FDA rather governs probiotic products within established paradigms based on their intended use, that is, as foods, food ingredients, dietary supplements, or drugs. Each of these has its own standards regarding safety, claims, efficacy, and manufacturing practices [50]. Under the current system, probiotics marketed as dietary supplements are treated as “foods” and must comply with the Dietary Supplement Health and Education Act (DSHEA) as well as FDA guidelines. If a probiotic is intended for a medical therapeutic purpose, it is being treated as a “drug” and must undergo the formal FDA drug approval process. Probiotics used as biological products require authorization through a Biologic License Application (BLA) under FDA regulation [162]. In addition to product classification, the FDA also monitors the types of claims permitted for traditional probiotic foods and beverages. Although this is rare, health claims need to be pre-approved by the FDA through a formal petitioning process. However, structure, function, or nutrient content claims can be made without premarket approval, though companies are required by the FDA to provide notice to the agency 30 days before marketing. To make such claims viable, the Federal Trade Commission (FTC) requires evidence based on proper research and testing, which the FDA adopts to ensure that the claims are true and supported by good scientific research [162].

To augment the evidence base, the National Institutes of Health (NIH) and the FDA approved thorough systematic reviews in the auspices of the Agency for Healthcare Research and Quality (AHRQ). This review revolves around evaluating the safety profiles of the most used probiotic genera such as *Lactobacillus*, *Bifidobacterium*, *Saccharomyces*, *Streptococcus*, *Enterococcus*, and *Bacillus*, with special emphasis on when used for prevention, management, or reduction in disease risk [72,163]. The objective is to chart what is currently known about probiotic safety; determine the gaps; and provide guidance to practitioners, researchers, and regulators on best practices for assessing probiotic risk and benefit. Remarkably, early findings suggest that while many studies focus on probiotic efficacy, relatively few comprehensively address safety endpoints in sufficient detail [72]. This suggests the need for more systematic safety testing in future clinical trials to build strong regulatory agreement about what safety measures need to be standardized for probiotics in the American marketplace.

Collectively, these initiatives illustrate both the progress and persistent challenges in ensuring the safe use of probiotics, especially for novel and emerging strains. While there is agreement on core safety principles such as robust molecular identification, accurate functional screening, transparent strain documentation, and human trial data, the lack of harmonized global standards and clear, practical testing protocols remains a barrier. As the field of probiotics research continues to advance, it is crucial to align these efforts and close any gaps to balance innovation with consumer protection.

### 7.2. Research Gaps and Future Directions

By definition, a probiotic must deliver proven health benefit to the host. To meet this standard, there must be evidence of at least one rigorously performed human trial with an acceptable benefit on a relevant health outcome, ideally confirmed by follow-up studies of similar quality [10]. While there have been some promising findings, most of the probiotic research to date relies on short-term trials involving small sample sizes and insufficient follow-up, making it difficult to draw definitive conclusions about long-term safety, reliability, and real-world benefits. Moreover, demonstrating a true health benefit also depends not only on whether a trial is designed and conducted well, but on clear reporting and, ultimately, the ability of the scientific community to critically review results [2]. Therefore, the future generation of probiotic research must prioritize large-scale, randomized controlled trials that are methodologically rigorous, well reported, and for long durations. Such evidence is required to validate strain-specific benefit, establish dose–response, and rule out potential adverse effects with long-term application; all of which are critical to convincingly position probiotics as effective functional food ingredients backed by sound science.

As previously described, it is well recognized that probiotic benefits are highly strain-specific, with wide genetic differences between two probiotic strains of the same species [45]. However, this variability has broader implications for the functional classification of probiotics. For example, Douillard et al. [164] compared 100 isolates of *L. rhamnosus* from human and dairy origin and found that the strains displayed significant variations in essential functional traits such as resistance to bile acids, metabolism of carbohydrates, and possession of mucus-binding pili. This demonstrates that taxonomic classification alone is not an efficient predictor of the way a probiotic will perform; rather, the distinct functional attributes of each strain must be rigorously tested [2,45]. Hence, probiotic research cannot be limited to physiological classification, but each strain must be identified correctly, thoroughly characterized, and chosen based on both its genetic attributes, good evidence of its functional characteristics and its health impacts. It is this more nuanced understanding that constitutes the foundation of “precision probiotics” (strains that are selectively designed or bioengineered to produce predictable, specific effects in specific states of health).

Future studies must enhance the effort to define these strain-specific mechanisms of action and test the interaction of different strains with each other and the host in vivo. A deeper understanding is also needed on how probiotics and the human body interact, which include the immune system, gut barrier, and resident microbiota. While in vitro and in vivo studies have provided valuable information, more human-centric research will be required to map these interactions at the cellular and molecular levels and delineate with certainty how they deliver real health outcomes. Insight into these dynamic microbiome–host interactions could allow for the development of next-generation probiotics that specifically target the gut microbiome with more precision and deliver consistent, quantifiable health benefits. This also provides the foundation to explore multi-strain or synbiotic products designed to have synergistic or complementary effects. With the advancement in next-generation genomics, transcriptomics, and functional screening tools, precision probiotics will play a leading role in next-generation functional foods designed to meet individualized health requirements with scientific accuracy.

## 8. Conclusions

The growing global interest in functional foods continues to drive the discovery and application of emerging probiotic strains from unconventional sources such as fruits, vegetables, marine environments, etc. Different studies have demonstrated that these sources are valuable reservoirs of viable LAB and yeast species that contain unique techno-functional attributes. These emerging strains not only exhibit significant probiotic characteristics such as resistance to acid and bile, pathogen inhibition, and survival in the gut but also hold greater promise for additional health benefits such as anti-hypertensive, cholesterol-lowering, immunomodulatory, and anti-diabetic effects. However, to maximize the potential of these novel probiotic sources, it is necessary that every probable candidate strain is thoroughly screened and properly characterized, both phenotypically and genotypically. Care must be taken to verify safety attributes, including absence of hemolysis, no breakdown of mucin, and absence of transmissible antibiotic resistance genes, amongst other attributes that must be demonstrated and not assumed. Furthermore, while most of these novel strains have shown promising functional outcomes both in vitro and in animal models, robust human clinical trials must be performed to validate these positive effects across diverse groups such as the elderly, those with metabolic disorders, or individuals with specific dietary restrictions such as lactose intolerance. The integration of new technologies such as advanced molecular methods, culture-independent techniques, and omics-based technologies will augment the cataloguing of strain-specific effects, enabling specific probiotic development towards health outcomes. Importantly, to foster consumer trust, increased transparent regulatory guidance, labeling, and consumer education will be required as these emerging microbial strains are introduced into commercial markets. The future of probiotics in functional foods lies in exploring the wealth of microbial diversity in nature beyond the conventional dairy-based sources, and collaborative efforts are needed to drive this innovation for the benefit of global health and well-being.

## Figures and Tables

**Table 1:** Representative conventional and emerging probiotic species, their isolation sources, and key functional properties.

Species/Strains	Isolation Source	Key Functional Traits	References
*Weissella confusa* MD1 and *Weissella cibaria* MD2	Fermented batter, traditional fermented foods such as horreh	Co-aggregation with pathogens; lysozyme and acid tolerance; cholesterol reduction; antimicrobial potential; exopolysaccharide production; antioxidant activity.	[22,23]
*Bacillus coagulans*	Milk	Spore-forming abilities, gastrointestinal survivability; enzyme production; heat tolerance.	[24]
*Lactobacillus plantarum* (e.g., SY11, SY12, AAS3)	Kimchi, dry fish based fermented food	Acid and bile tolerance; adhesion to intestinal cells; cholesterol reducing property; antimicrobial and antioxidant activity.	[25,26]
*L*. *paraplantarum* (e.g., SC61)	Jangajii (fermented vegetable)	Antioxidant and immunostimulatory activity; stability in artificial gastric and bile conditions, non-production of β-glucuronidase, suitable antibiotic susceptibility, and attachment to intestinal cells.	[27]
*Weissella hellenica* BCC 7239	Nham (fermented pork sausage)	Production of bacteriocins, bactericidal effects against both Gram-positive and Gram-negative organisms	[28]
*Fructobacillus fructosus* MCC 3996	Flower nectar	Resistance to gastric conditions; co-aggregation with pathogens, hydrophobicity, and the absence of hemolytic activity.	[29]
*Saccharomyces cerevisiae* (e.g., KU200270, KU200280, and KU200284)	Cucumber *jangajji* and other fermented foods	Antioxidative properties; gastric and bile resistance; adhesion to epithelial cells.	[30]
*Aureobasidium pullulans* (e.g., Y39, Y40, Y41, Y43)	Kalamata table olive	Auto-aggregation ability; hydrophobicity; adhesion to Caco-2 cells; absence of hemolytic activity.	[31]
*Lacticaseibacillus casei* (e.g., SB71, SB73 and SB93)	Marine ecosystem	Inability to form biogenic amines; adherence to Caco-2 cells; cholesterol assimilation; and tolerance to NaCl, bile and low pH.	[32]
*Leuconostoc* (*citreum* and *mesenteroides* subsp. *mesenteroides*)	Traditional fermented foods such as Horreh	Exopolysaccharide production; antioxidant activity; acid tolerance	[23]
*Pediococcus* *pentosaceus*	Traditional fermented foods	Exopolysaccharide production; antioxidant activity; acid tolerance	[23]
*Enterococcus* (*faecium* and *faecalis*)	Traditional fermented foods (e.g., Kimchi, Horreh)	Absence of antibiotic resistance or virulence factors; auto aggregation ability; hydrophobicity; resistance to gastrointestinal conditions.	[23,33]
*Akkermansia muciniphila*	Human intestinal microbiota	Mucin degradation; modulation of host metabolism; gut barrier reinforcement	[34]
*Faecalibacterium prausnitzii*	Human gut	Butyrate production; anti-inflammatory and gut-protective effects	[35]

**Table 2:** Conceptual Comparison between Conventional Probiotics and Emerging Probiotic Candidates.

Criteria	Conventional Probiotics (e.g., *Lactobacillus*, *Bifidobacterium*)	Emerging Probiotics (e.g., *Akkermansia muciniphila, Faecalibacterium prausnitzii*)
Safety status	Well-established (GRAS/QPS) [2,10]	Ongoing safety evaluation and limited regulatory approval [21]
Isolation sources	Traditional fermented foods (yogurt, kefir, sauerkraut), dairy products, and human/animal gut [5,6]	Novel environments (soil, plants, insects, marine microbiota, human gut) [21,32,35]
Health benefits (evidence base)	Well-documented gut health and anti-diarrheal effects, lactose intolerance relief; multiple clinical trials [5,10]	Limited but growing number of clinical studies; promising roles in obesity, diabetes, inflammatory bowel disease, and metabolic syndrome [42]
Functional traits	Antimicrobial activity; acid and bile salt tolerance, epithelial adhesion [25,26]	Immune modulation, gut barrier enhancement, production of short-chain fatty acid (SCFA) [43,44]
Mechanistic understanding	Mechanisms relatively well-characterized (competition with pathogens, production of antimicrobials, adhesion, immune modulation) [2].	Mechanisms still being evaluated (mucin degradation, signaling via metabolites like SCFAs, anti-inflammatory pathways) [42,44]
Industrial application	Widely commercialized in yogurts, cheeses, beverages, infant formula, dietary supplements [38].	Limited commercial applications; potential in next-generation probiotics (capsules, synbiotics, functional beverages) [42]
Challenges	Strain-specific variability; genetic instability in industrial settings [45]	Difficulties in cultivation, safety uncertainties, lack of regulatory approval, stability issues in food matrices [21]
Future perspectives	Continued use in conventional foods and nutraceuticals; exploration of strain engineering for enhanced traits	Potential game-changers in personalized nutrition, microbiome-targeted therapies, and precision probiotics once validated

## Data Availability

No new data were created or analyzed in this study. Data sharing is not applicable to this article.

## References

[1] Birch C.S., Bonwick G.A. (2019). Ensuring the future of functional foods. Int. J. Food Sci. Technol..

[2] Binda S., Hill C., Johansen E., Obis D., Pot B., Sanders M.E., Tremblay A., Ouwehand A.C. (2020). Criteria to qualify microorganisms as “probiotic” in foods and dietary supplements. Front. Microbiol..

[3] Shimizu M. (2019). History and current status of functional food regulations in Japan. Nutraceutical and Functional Food Regulations in the United States and Around the World.

[4] Manach C., Milenkovic D., Van de Wiele T., Rodriguez-Mateos A., de Roos B., Garcia-Conesa M.T., Landberg R., Gibney E.R., Heinonen M., Tomás-Barberán F. (2017). Addressing the inter-individual variation in response to consumption of plant food bioactives: Towards a better understanding of their role in healthy aging and cardiometabolic risk reduction. Mol. Nutr. Food Res..

[5] Dong Y., Li M., Yue X. (2024). Current research on probiotics and fermented products. Foods.

[6] Oyeniran A., Gyawali R., Aljaloud S.O., Krastanov A., Ibrahim S.A. (2020). Probiotic characteristics and health benefits of the yogurt bacterium Lactobacillus delbrueckii sp. bulgaricus. Current Issues and Challenges in the Dairy Industry.

[7] Khan R.S., Grigor J., Winger R., Win A. (2013). Functional food product development–Opportunities and challenges for food manufacturers. Trends Food Sci. Technol..

[8] Vlaicu P.A., Untea A.E., Varzaru I., Saracila M., Oancea A.G. (2023). Designing nutrition for health—Incorporating dietary by-products into poultry feeds to create functional foods with insights into health benefits, risks, bioactive compounds, food component functionality and safety regulations. Foods.

[9] Latif A., Shehzad A., Niazi S., Zahid A., Ashraf W., Iqbal M.W., Rehman A., Riaz T., Aadil R.M., Khan I.M. (2023). Probiotics: Mechanism of action, health benefits and their application in food industries. Front. Microbiol..

[10] Hill C., Guarner F., Reid G., Gibson G.R., Merenstein D.J., Pot B., Morelli L., Canani R.B., Flint H.J., Salminen S. (2014). Expert consensus document: The International Scientific Association for Probiotics and Prebiotics consensus statement on the scope and appropriate use of the term probiotic. Nat. Rev. Gastroenterol. Hepatol..

[11] ISAPP (2018). Minimum Criteria for Probiotics [Online].

[12] Champagne C.P., da Cruz A.G., Daga M. (2018). Strategies to improve the functionality of probiotics in supplements and foods. Curr. Opin. Food Sci..

[13] Granato D., Branco G.F., Nazzaro F., Cruz A.G., Faria J.A. (2010). Functional foods and nondairy probiotic food development: Trends, concepts, and products. Compr. Rev. Food Sci. Food Saf..

[14] Corbo M.R., Campaniello D., Speranza B., Altieri C., Sinigaglia M., Bevilacqua A. (2018). Neutralisation of toxins by probiotics during the transit into the gut: Challenges and perspectives. Int. J. Food Sci. Technol..

[15] Mokhtari S., Khomeiri M., Jafari S.M., Maghsoudlou Y., Ghorbani M. (2017). Descriptive analysis of bacterial profile, physicochemical and sensory characteristics of grape juice containing *Saccharomyces cerevisiae* cell wall-coated probiotic microcapsules during storage. Int. J. Food Sci. Technol..

[16] Campaniello D., Speranza B., Petruzzi L., Bevilacqua A., Corbo M.R. (2018). How to routinely assess transition, adhesion and survival of probiotics into the gut: A case study on propionibacteria. Int. J. Food Sci. Technol..

[17] Moumita S., Das B., Sundaray A., Satpathi S., Thangaraj P., Marimuthu S., Jayabalan R. (2018). Study of soy-fortified green tea curd formulated using potential hypocholesterolemic and hypotensive probiotics isolated from locally made curd. Food Chem..

[18] Mohammadi-Sartang M., Bellissimo N., de Zepetnek J.T., Brett N., Mazloomi S., Fararouie M., Bedeltavana A., Famouri M., Mazloom Z. (2018). The effect of daily fortified yogurt consumption on weight loss in adults with metabolic syndrome: A 10-week randomized controlled trial. Nutr. Metab. Cardiovasc. Dis..

[19] Ephrem E., Najjar A., Charcosset C., Greige-Gerges H. (2018). Encapsulation of natural active compounds, enzymes, and probiotics for fruit juice fortification, preservation, and processing: An overview. J. Funct. Foods.

[20] Wardill H.R., Van Sebille Y.Z., Ciorba M.A., Bowen J.M. (2018). Prophylactic probiotics for cancer therapy-induced diarrhoea: A meta-analysis. Curr. Opin. Support. Palliat. Care.

[21] Pimentel T.C., de Oliveira L.I.G., Macedo E.d.L.C., Costa G.N., Dias D.R., Schwan R.F., Magnani M. (2021). Understanding the potential of fruits, flowers, and ethnic beverages as valuable sources of techno-functional and probiotics strains: Current scenario and main challenges. Trends Food Sci. Technol..

[22] Lakra A.K., Domdi L., Hanjon G., Tilwani Y.M., Arul V. (2020). Some probiotic potential of *Weissella confusa* MD1 and *Weissella cibaria* MD2 isolated from fermented batter. LWT.

[23] Vasiee A., Alizadeh Behbahani B., Tabatabaei Yazdi F., Mortazavi S.A., Noorbakhsh H. (2018). Diversity and probiotic potential of lactic acid bacteria isolated from horreh, a traditional Iranian fermented food. Probiotics Antimicrob. Proteins.

[24] Cao J., Yu Z., Liu W., Zhao J., Zhang H., Zhai Q., Chen W. (2020). Probiotic characteristics of *Bacillus coagulans* and associated implications for human health and diseases. J. Funct. Foods.

[25] Lee N.-K., Kim S.-Y., Han K.J., Eom S.J., Paik H.-D. (2014). Probiotic potential of *Lactobacillus* strains with anti-allergic effects from kimchi for yogurt starters. LWT-Food Sci. Technol..

[26] Aarti C., Khusro A. (2019). Functional and technological properties of exopolysaccharide producing autochthonous *Lactobacillus plantarum* strain AAS3 from dry fish based fermented food. LWT.

[27] Son S.-H., Yang S.-J., Jeon H.-L., Yu H.-S., Lee N.-K., Park Y.-S., Paik H.-D. (2018). Antioxidant and immunostimulatory effect of potential probiotic Lactobacillus paraplantarum SC61 isolated from Korean traditional fermented food, jangajji. Microb. Pathog..

[28] Woraprayote W., Pumpuang L., Tosukhowong A., Roytrakul S., Perez R.H., Zendo T., Sonomoto K., Benjakul S., Visessanguan W. (2015). Two putatively novel bacteriocins active against Gram-negative food borne pathogens produced by *Weissella hellenica* BCC 7293. Food Control.

[29] Patil M., Jadhav A., Patil U. (2020). Functional characterization and in vitro screening of *Fructobacillus fructosus* MCC 3996 isolated from *Butea monosperma* flower for probiotic potential. Lett. Appl. Microbiol..

[30] Lee N.-K., Hong J.-Y., Yi S.-H., Hong S.-P., Lee J.-E., Paik H.-D. (2019). Bioactive compounds of probiotic *Saccharomyces cerevisiae* strains isolated from cucumber jangajji. J. Funct. Foods.

[31] Bonatsou S., Karamouza M., Zoumpopoulou G., Mavrogonatou E., Kletsas D., Papadimitriou K., Tsakalidou E., Nychas G.-J.E., Panagou E.Ζ. (2018). Evaluating the probiotic potential and technological characteristics of yeasts implicated in cv. Kalamata natural black olive fermentation. Int. J. Food Microbiol..

[32] Das P., Khowala S., Biswas S. (2016). In vitro probiotic characterization of *Lactobacillus casei* isolated from marine samples. LWT.

[33] Kim Y., Choi S.-I., Jeong Y., Kang C.-H. (2022). Evaluation of safety and probiotic potential of *Enterococcus faecalis* MG5206 and *Enterococcus faecium* MG5232 isolated from kimchi, a Korean fermented cabbage. Microorganisms.

[34] Zhang T., Li Q., Cheng L., Buch H., Zhang F. (2019). Akkermansia muciniphila is a promising probiotic. Microb. Biotechnol..

[35] Martín R., Miquel S., Benevides L., Bridonneau C., Robert V., Hudault S., Chain F., Berteau O., Azevedo V., Chatel J.M. (2017). Functional characterization of novel *Faecalibacterium prausnitzii* strains isolated from healthy volunteers: A step forward in the use of *F. prausnitzii* as a next-generation probiotic. Front. Microbiol..

[36] Brodmann T., Endo A., Gueimonde M., Vinderola G., Kneifel W., de Vos W.M., Salminen S., Gómez-Gallego C. (2017). Safety of novel microbes for human consumption: Practical examples of assessment in the European Union. Front. Microbiol..

[37] Bubnov R.V., Babenko L.P., Lazarenko L.M., Mokrozub V.V., Spivak M.Y. (2018). Specific properties of probiotic strains: Relevance and benefits for the host. EPMA J..

[38] García-Ruiz A., de Llano D.G., Esteban-Fernández A., Requena T., Bartolomé B., Moreno-Arribas M.V. (2014). Assessment of probiotic properties in lactic acid bacteria isolated from wine. Food Microbiol..

[39] Salmerón I. (2017). Fermented cereal beverages: From probiotic, prebiotic and synbiotic towards Nanoscience designed healthy drinks. Lett. Appl. Microbiol..

[40] Gupta S., Abu-Ghannam N. (2012). Probiotic fermentation of plant based products: Possibilities and opportunities. Crit. Rev. Food Sci. Nutr..

[41] Betoret N., Puente L., Dıaz M., Pagán M., Garcıa M., Gras M., Martínez-Monzó J., Fito P. (2003). Development of probiotic-enriched dried fruits by vacuum impregnation. J. Food Eng..

[42] Verhoog S., Taneri P.E., Roa Díaz Z.M., Marques-Vidal P., Troup J.P., Bally L., Franco O.H., Glisic M., Muka T. (2019). Dietary factors and modulation of bacteria strains of *Akkermansia muciniphila* and *Faecalibacterium prausnitzii*: A systematic review. Nutrients.

[43] Pabari K., Pithva S., Kothari C., Purama R.K., Kondepudi K.K., Vyas B.R.M., Kothari R., Ambalam P. (2020). Evaluation of probiotic properties and prebiotic utilization potential of Weissella paramesenteroides isolated from fruits. Probiotics Antimicrob. Proteins.

[44] Mei L., Wang J., Hao Y., Zeng X., Yang Y., Wu Z., Ji Y. (2024). A comprehensive update on the immunoregulatory mechanisms of *Akkermansia muciniphila*: Insights into active ingredients, metabolites, and nutrient-driven modulation. Crit. Rev. Food Sci. Nutr..

[45] Day R.L., Harper A.J., Woods R.M., Davies O.G., Heaney L.M. (2019). Probiotics: Current landscape and future horizons. Future Sci. OA.

[46] FAO/WHO (2002). Guidelines for the evaluation of probiotics in food. Food and Agriculture Organization of the United Nations and World Health Organization Working Group Report.

[47] de Albuquerque T.M.R., Garcia E.F., de Oliveira Araújo A., Magnani M., Saarela M., de Souza E.L. (2018). In vitro characterization of Lactobacillus strains isolated from fruit processing by-products as potential probiotics. Probiotics Antimicrob. Proteins.

[48] Wattam A.R., Davis J.J., Assaf R., Boisvert S., Brettin T., Bun C., Conrad N., Dietrich E.M., Disz T., Gabbard J.L. (2017). Improvements to PATRIC, the all-bacterial bioinformatics database and analysis resource center. Nucleic Acids Res..

[49] Kõll-Klais P., Mändar R., Leibur E., Marcotte H., Hammarström L., Mikelsaar M. (2005). Oral lactobacilli in chronic periodontitis and periodontal health: Species composition and antimicrobial activity. Oral Microbiol. Immunol..

[50] de Melo Pereira G.V., de Oliveira Coelho B., Júnior A.I.M., Thomaz-Soccol V., Soccol C.R. (2018). How to select a probiotic? A review and update of methods and criteria. Biotechnol. Adv..

[51] Xu Y., Zhou T., Tang H., Li X., Chen Y., Zhang L., Zhang J. (2020). Probiotic potential and amylolytic properties of lactic acid bacteria isolated from Chinese fermented cereal foods. Food Control.

[52] Garcia E.F., Luciano W.A., Xavier D.E., da Costa W.C., de Sousa Oliveira K., Franco O.L., de Morais Junior M.A., Lucena B.T., Picao R.C., Magnani M. (2016). Identification of lactic acid bacteria in fruit pulp processing byproducts and potential probiotic properties of selected *Lactobacillus* strains. Front. Microbiol..

[53] Fernández-Pacheco P., García-Béjar B., Jiménez-del Castillo M., Carreño-Domínguez J., Briones Pérez A., Arévalo-Villena M. (2021). Potential probiotic and food protection role of wild yeasts isolated from pistachio fruits (*Pistacia vera*). J. Sci. Food Agric..

[54] Maragkoudakis P.A., Zoumpopoulou G., Miaris C., Kalantzopoulos G., Pot B., Tsakalidou E. (2006). Probiotic potential of *Lactobacillus* strains isolated from dairy products. Int. Dairy J..

[55] Ogunremi O., Sanni A., Agrawal R. (2015). Probiotic potentials of yeasts isolated from some cereal-based Nigerian traditional fermented food products. J. Appl. Microbiol..

[56] Boonaert C.J., Rouxhet P.G. (2000). Surface of lactic acid bacteria: Relationships between chemical composition and physicochemical properties. Appl. Environ. Microbiol..

[57] Duary R.K., Rajput Y.S., Batish V.K., Grover S. (2011). Assessing the adhesion of putative indigenous probiotic lactobacilli to human colonic epithelial cells. Indian J. Med. Res..

[58] Tuo Y., Yu H., Ai L., Wu Z., Guo B., Chen W. (2013). Aggregation and adhesion properties of 22 *Lactobacillus* strains. J. Dairy Sci..

[59] Chelliah R., Ramakrishnan S.R., Prabhu P.R., Antony U. (2016). Evaluation of antimicrobial activity and probiotic properties of wild-strain *Pichia kudriavzevii* isolated from frozen idli batter. Yeast.

[60] Leite A.M., Miguel M., Peixoto R., Ruas-Madiedo P., Paschoalin V., Mayo B., Delgado S. (2015). Probiotic potential of selected lactic acid bacteria strains isolated from Brazilian kefir grains. J. Dairy Sci..

[61] Ramos C.L., Thorsen L., Schwan R.F., Jespersen L. (2013). Strain-specific probiotics properties of *Lactobacillus fermentum*, *Lactobacillus plantarum* and *Lactobacillus brevis* isolates from Brazilian food products. Food Microbiol..

[62] Mihaylova-Garnizova R., Davidova S., Hodzhev Y., Satchanska G. (2024). Antimicrobial peptides derived from bacteria: Classification, sources, and mechanism of action against multidrug-resistant bacteria. Int. J. Mol. Sci..

[63] Vera-Pingitore E., Jimenez M.E., Dallagnol A., Belfiore C., Fontana C., Fontana P., von Wright A., Vignolo G., Plumed-Ferrer C. (2016). Screening and characterization of potential probiotic and starter bacteria for plant fermentations. LWT-Food Sci. Technol..

[64] Divya J.B., Varsha K.K., Nampoothiri K.M. (2012). Newly isolated lactic acid bacteria with probiotic features for potential application in food industry. Appl. Biochem. Biotechnol..

[65] Inturri R., Stivala A., Furneri P., Blandino G. (2016). Growth and adhesion to HT-29 cells inhibition of Gram-negatives by *Bifidobacterium longum* BB536 e *Lactobacillus rhamnosus* HN001 alone and in combination. Eur. Rev. Med. Pharmacol. Sci..

[66] Benítez-Cabello A., Calero-Delgado B., Rodríguez-Gómez F., Garrido-Fernández A., Jiménez-Díaz R., Arroyo-López F.N. (2019). Biodiversity and multifunctional features of lactic acid bacteria isolated from table olive biofilms. Front. Microbiol..

[67] Sanders M.E., Shane A.L., Merenstein D.J. (2016). Advancing probiotic research in humans in the United States: Challenges and strategies. Gut Microbes.

[68] Yadav R., Shukla P. (2017). An overview of advanced technologies for selection of probiotics and their expediency: A review. Crit. Rev. Food Sci. Nutr..

[69] Bagheripoor-Fallah N., Mortazavian A., Hosseini H., Khoshgozaran-Abras S., Rad A.H. (2015). Comparison of molecular techniques with other methods for identification and enumeration of probiotics in fermented milk products. Crit. Rev. Food Sci. Nutr..

[70] Temmerman R., Huys G., Swings J. (2004). Identification of lactic acid bacteria: Culture-dependent and culture-independent methods. Trends Food Sci. Technol..

[71] Chen Y., Hsiao P., Hong W., Dai T., Chen M. (2012). Lactobacillus kefiranofaciens M1 isolated from milk kefir grains ameliorates experimental colitis in vitro and in vivo. J. Dairy Sci..

[72] Sanders M.E., Akkermans L.M., Haller D., Hammerman C., Heimbach J.T., Hörmannsperger G., Huys G. (2010). Safety assessment of probiotics for human use. Gut Microbes.

[73] Ashraf R., Shah N.P. (2011). Antibiotic resistance of probiotic organisms and safety of probiotic dairy products. Int. Food Res. J..

[74] Klare I., Konstabel C., Werner G., Huys G., Vankerckhoven V., Kahlmeter G., Hildebrandt B., Müller-Bertling S., Witte W., Goossens H. (2007). Antimicrobial susceptibilities of *Lactobacillus*, *Pediococcus* and *Lactococcus* human isolates and cultures intended for probiotic or nutritional use. J. Antimicrob. Chemother..

[75] Nawaz M., Wang J., Zhou A., Ma C., Wu X., Moore J.E., Cherie Millar B., Xu J. (2011). Characterization and transfer of antibiotic resistance in lactic acid bacteria from fermented food products. Curr. Microbiol..

[76] Senan S., Prajapati J., Joshi C. (2015). Feasibility of genome-wide screening for biosafety assessment of probiotics: A case study of Lactobacillus helveticus MTCC 5463. Probiotics Antimicrob. Proteins.

[77] Schulz K.F., Altman D.G., Moher D., The CONSORT Group (2010). CONSORT 2010 statement: Updated guidelines for reporting parallel group randomized trials. Ann. Intern. Med..

[78] Hutchinson A.N., Bergh C., Kruger K., Sűsserová M., Allen J., Améen S., Tingö L. (2021). The effect of probiotics on health outcomes in the elderly: A systematic review of randomized, placebo-controlled studies. Microorganisms.

[79] Zielińska D., Ołdak A., Rzepkowska A., Zieliński K. (2018). Enumeration and identification of probiotic bacteria in food matrices. Advances in Biotechnology for Food Industry.

[80] Sornplang P., Piyadeatsoontorn S. (2016). Probiotic isolates from unconventional sources: A review. J. Anim. Sci. Technol..

[81] Nanasombat S., Phunpruch S., Jaichalad T. (2012). Screening and identification of lactic acid bacteria from raw seafoods and Thai fermented seafood products for their potential use as starter cultures. Songklanakarin J. Sci. Technol..

[82] Senthong R., Chanthachum S., Sumpavapol P. Screening and identification of probiotic lactic acid bacteria isolated from Poo-Khem, A traditional salted crab. Proceedings of the International Conference on Nutrition and Food Sciences.

[83] Miyashita M., Yukphan P., Chaipitakchonlatarn W., Malimas T., Sugimoto M., Yoshino M., Potacharoen W., Tanasupawat S., Nakagawa Y., Kirtikara K. (2012). 16S rRNA gene sequence analysis of lactic acid bacteria isolated from fermented foods in Thailand. Microbiol. Cult. Coll..

[84] Siripornadulsil W., Tasaku S., Buahorm J., Siripornadulsil S. (2014). Probiotic properties of lactic acid bacteria isolated from fermented food. Intl. J. Biol. Food Vet. Agri. Eng..

[85] Bacha K., Mehari T., Ashenafi M. (2009). In-vitro probiotic potential of lactic acid bacteria isolated from ‘Wakalim’, a traditional Ethiopian fermented beef sausage. Ethiop. J. Health Sci..

[86] Agaliya P.J., Jeevaratnam K. (2012). Screening of Lactobacillus plantarum isolated from fermented idli batter for probiotic properties. Afr. J. Biotechnol..

[87] Oluwajoba S.O., Akinyosoye F.A., Oyetayo V.O. (2013). In vitro screening and selection of probiotic lactic acid bacteria isolated from spontaneously fermenting Kunu-Zaki. Adv. Microbiol..

[88] Sarıtaş S., Mondragon Portocarrero A.d.C., Miranda J.M., Witkowska A.M., Karav S. (2024). Functional Yogurt: Types and Health Benefits. Appl. Sci..

[89] Sharifi Yazdi M.K., Davoodabadi A., Khesht Zarin H.R., Tajabadi Ebrahimi M., Soltan Dallal M.M. (2017). Characterisation and probiotic potential of lactic acid bacteria isolated from Iranian traditional yogurts. Ital. J. Anim. Sci..

[90] Anumudu C.K., Ikimi C.G., Zige D.V., Omeje F.I., Gbodo E.E. (2019). Production of Bacteriocins by *Lactobacillus plantarum* and Pediococcus acidilactici Isolated from Cow Milk. Niger. J. Microbiol..

[91] Hoque M., Akter F., Hossain K., Rahman M., Billah M., Islam K. (2010). Isolation, identification and analysis of probiotic properties of *Lactobacillus* spp. from selective regional yoghurts. World J. Dairy Food Sci..

[92] Plessas S., Nouska C., Mantzourani I., Kourkoutas Y., Alexopoulos A., Bezirtzoglou E. (2017). Microbiological exploration of different types of kefir grains. Fermentation.

[93] Diosma G., Romanin D.E., Rey-Burusco M.F., Londero A., Garrote G.L. (2014). Yeasts from kefir grains: Isolation, identification, and probiotic characterization. World J. Microbiol. Biotechnol..

[94] Maeno S., Kajikawa A., Dicks L., Endo A. (2019). Introduction of bifunctional alcohol/acetaldehyde dehydrogenase gene (adhE) in *Fructobacillus fructosus* settled its fructophilic characteristics. Res. Microbiol..

[95] Behare P.V., Mazhar S., Pennone V., McAuliffe O. (2020). Evaluation of lactic acid bacteria strains isolated from fructose-rich environments for their mannitol-production and milk-gelation abilities. J. Dairy Sci..

[96] Sakandar H.A., Kubow S., Sadiq F.A. (2019). Isolation and in-vitro probiotic characterization of fructophilic lactic acid bacteria from Chinese fruits and flowers. Lwt.

[97] Adetunji C.O., Akram M., Michael O.S., Shahzad K., Ayeni A.E., Hasan S., Adetunji J.B., Hasan S.M., Inamuddin, Olaniyan M. (2021). Polysaccharides derived from natural sources: A panacea to health and nutritional challenges. Polysaccharides: Properties and Applications.

[98] Rodrigues N.P.A., Garcia E.F., de Souza E.L. (2021). Selection of lactic acid bacteria with promising probiotic aptitudes from fruit and ability to survive in different food matrices. Braz. J. Microbiol..

[99] Tenea G.N., Perugachi E. (2025). Innovative functional juice enriched with native probiotics for enhanced nutrition and antimicrobial properties. Front. Nutr..

[100] Sarao L.K., Arora M. (2017). Probiotics, prebiotics, and microencapsulation: A review. Crit. Rev. Food Sci. Nutr..

[101] WGO (2017). Global Guidelines: Probiotics and Prebiotics.

[102] Flach J., van der Waal M.B., van den Nieuwboer M., Claassen E., Larsen O.F. (2018). The underexposed role of food matrices in probiotic products: Reviewing the relationship between carrier matrices and product parameters. Crit. Rev. Food Sci. Nutr..

[103] Yao M., Xie J., Du H., McClements D.J., Xiao H., Li L. (2020). Progress in microencapsulation of probiotics: A review. Compr. Rev. Food Sci. Food Saf..

[104] Reque P.M., Brandelli A. (2021). Encapsulation of probiotics and nutraceuticals: Applications in functional food industry. Trends Food Sci. Technol..

[105] Fenster K., Freeburg B., Hollard C., Wong C., Rønhave Laursen R., Ouwehand A.C. (2019). The production and delivery of probiotics: A review of a practical approach. Microorganisms.

[106] Terpou A., Papadaki A., Lappa I.K., Kachrimanidou V., Bosnea L.A., Kopsahelis N. (2019). Probiotics in food systems: Significance and emerging strategies towards improved viability and delivery of enhanced beneficial value. Nutrients.

[107] Singh S., Gupta R., Chawla S., Gauba P., Singh M., Tiwari R.K., Upadhyay S., Sharma S., Chanda S., Gaur S. (2022). Natural sources and encapsulating materials for probiotics delivery systems: Recent applications and challenges in functional food development. Front. Nutr..

[108] Ta L.P., Bujna E., Antal O., Ladányi M., Juhász R., Szécsi A., Kun S., Sudheer S., Gupta V.K., Nguyen Q.D. (2021). Effects of various polysaccharides (alginate, carrageenan, gums, chitosan) and their combination with prebiotic saccharides (resistant starch, lactosucrose, lactulose) on the encapsulation of probiotic bacteria Lactobacillus casei 01 strain. Int. J. Biol. Macromol..

[109] Anumudu C., Miri T., Onyeaka H. (2024). Sporicidal Activity of Micro-Encapsulated Nisin-like Bacteriocins Obtained from *Lactococcus lactis* against *Bacillus cereus* spores. Proceedings of the 2024 European Symposium on Food Safety.

[110] Heunis T., Botes M., Dicks L. (2010). Encapsulation of *Lactobacillus plantarum* 423 and its bacteriocin in nanofibers. Probiotics Antimicrob. Proteins.

[111] Afzaal M., Khan A.U., Saeed F., Ahmed A., Ahmad M.H., Maan A.A., Tufail T., Anjum F.M., Hussain S. (2019). Functional exploration of free and encapsulated probiotic bacteria in yogurt and simulated gastrointestinal conditions. Food Sci. Nutr..

[112] Chen L., Yang T., Song Y., Shu G., Chen H. (2017). Effect of xanthan-chitosan-xanthan double layer encapsulation on survival of Bifidobacterium BB01 in simulated gastrointestinal conditions, bile salt solution and yogurt. LWT-Food Sci. Technol..

[113] Dias C.O., de Almeida J.d.S.O., Pinto S.S., de Oliveira Santana F.C., Verruck S., Müller C.M.O., Prudêncio E.S., Amboni R.D.d.M.C. (2018). Development and physico-chemical characterization of microencapsulated bifidobacteria in passion fruit juice: A functional non-dairy product for probiotic delivery. Food Biosci..

[114] Mokhtari S., Jafari S.M., Khomeiri M. (2019). Survival of encapsulated probiotics in pasteurized grape juice and evaluation of their properties during storage. Food Sci. Technol. Int..

[115] Asar R., Erenler S., Devecioglu D., Ispirli H., Karbancioglu-Guler F., Ozturk H.I., Dertli E. (2025). Understanding the Functionality of Probiotics on the Edge of Artificial Intelligence (AI) Era. Fermentation.

[116] Westfall S., Carracci F., Estill M., Zhao D., Wu Q.-l., Shen L., Simon J., Pasinetti G.M. (2021). Optimization of probiotic therapeutics using machine learning in an artificial human gastrointestinal tract. Sci. Rep..

[117] Sadeghi M., Panahi B., Mazlumi A., Hejazi M.A., Komi D.E.A., Nami Y. (2022). Screening of potential probiotic lactic acid bacteria with antimicrobial properties and selection of superior bacteria for application as biocontrol using machine learning models. LWT.

[118] Zhang D., Zhang J., Kalimuthu S., Liu J., Song Z.-M., He B.-b., Cai P., Zhong Z., Feng C., Neelakantan P. (2023). A systematically biosynthetic investigation of lactic acid bacteria reveals diverse antagonistic bacteriocins that potentially shape the human microbiome. Microbiome.

[119] van Heel A.J., de Jong A., Song C., Viel J.H., Kok J., Kuipers O.P. (2018). BAGEL4: A user-friendly web server to thoroughly mine RiPPs and bacteriocins. Nucleic Acids Res..

[120] Arango-Argoty G., Garner E., Pruden A., Heath L.S., Vikesland P., Zhang L. (2018). DeepARG: A deep learning approach for predicting antibiotic resistance genes from metagenomic data. Microbiome.

[121] Krawczyk P.S., Lipinski L., Dziembowski A. (2018). PlasFlow: Predicting plasmid sequences in metagenomic data using genome signatures. Nucleic Acids Res..

[122] Wu S., Feng T., Tang W., Qi C., Gao J., He X., Wang J., Zhou H., Fang Z. (2024). metaProbiotics: A tool for mining probiotic from metagenomic binning data based on a language model. Brief. Bioinform..

[123] González-Sendino R., Serrano E., Bajo J. (2024). Mitigating bias in artificial intelligence: Fair data generation via causal models for transparent and explainable decision-making. Future Gener. Comput. Syst..

[124] D’Urso F., Broccolo F. (2024). Applications of artificial intelligence in microbiome analysis and probiotic interventions—An overview and perspective based on the current state of the art. Appl. Sci..

[125] Song M.W., Chung Y., Kim K.-T., Hong W.S., Chang H.J., Paik H.-D. (2020). Probiotic characteristics of *Lactobacillus brevis* B13-2 isolated from kimchi and investigation of antioxidant and immune-modulating abilities of its heat-killed cells. LWT.

[126] Fakruddin M., Hossain M.N., Ahmed M.M. (2017). Antimicrobial and antioxidant activities of Saccharomyces cerevisiae IFST062013, a potential probiotic. BMC Complement. Altern. Med..

[127] Ornellas R.M.S., Santos T.T., Arcucio L.B., Sandes S.H.C., Oliveira M.M., Dias C.V., de Carvalho Silva S., Uetanabaro A.P.T., Vinderola G., Nicoli J.R. (2017). Selection of lactic acid bacteria with probiotic potential isolated from the fermentation process of “Cupuaçu”(*Theobroma grandiflorum*). Adv. Microbiol. Infect. Dis. Public Health.

[128] Ooi L.-G., Liong M.-T. (2010). Cholesterol-lowering effects of probiotics and prebiotics: A review of in vivo and in vitro findings. Int. J. Mol. Sci..

[129] Kumar M., Nagpal R., Kumar R., Hemalatha R., Verma V., Kumar A., Chakraborty C., Singh B., Marotta F., Jain S. (2012). Cholesterol-lowering probiotics as potential biotherapeutics for metabolic diseases. J. Diabetes Res..

[130] Cavalcante R.G., de Albuquerque T.M., de Luna Freire M.O., Ferreira G.A., Dos Santos L.A.C., Magnani M., Cruz J.C., Braga V.A., de Souza E.L., de Brito Alves J.L. (2019). The probiotic Lactobacillus fermentum 296 attenuates cardiometabolic disorders in high fat diet-treated rats. Nutr. Metab. Cardiovasc. Dis..

[131] Costabile A., Buttarazzi I., Kolida S., Quercia S., Baldini J., Swann J.R., Brigidi P., Gibson G.R. (2017). An in vivo assessment of the cholesterol-lowering efficacy of *Lactobacillus plantarum* ECGC 13110402 in normal to mildly hypercholesterolaemic adults. PLoS ONE.

[132] Tannock G.W., Savage D.C. (1974). Influences of dietary and environmental stress on microbial populations in the murine gastrointestinal tract. Infect. Immun..

[133] Luna R.A., Foster J.A. (2015). Gut brain axis: Diet microbiota interactions and implications for modulation of anxiety and depression. Curr. Opin. Biotechnol..

[134] Collins S.M., Surette M., Bercik P. (2012). The interplay between the intestinal microbiota and the brain. Nat. Rev. Microbiol..

[135] Dinan T.G., Cryan J.F. (2013). Melancholic microbes: A link between gut microbiota and depression?. Neurogastroenterol. Motil..

[136] Akkasheh G., Kashani-Poor Z., Tajabadi-Ebrahimi M., Jafari P., Akbari H., Taghizadeh M., Memarzadeh M.R., Asemi Z., Esmaillzadeh A. (2016). Clinical and metabolic response to probiotic administration in patients with major depressive disorder: A randomized, double-blind, placebo-controlled trial. Nutrition.

[137] Kouitcheu Mabeku L.B., Ngue S., Bonsou Nguemo I., Leundji H. (2020). Potential of selected lactic acid bacteria from *Theobroma cacao* fermented fruit juice and cell-free supernatants from cultures as inhibitors of Helicobacter pylori and as good probiotic. BMC Res. Notes.

[138] Di Cagno R., Filannino P., Cantatore V., Polo A., Celano G., Martinovic A., Cavoski I., Gobbetti M. (2020). Design of potential probiotic yeast starters tailored for making a cornelian cherry (*Cornus mas* L.) functional beverage. Int. J. Food Microbiol..

[139] Sardar D., Morol I., Bari J., Sarkar A., Habib A. (2025). Optimization of cryoprotectants and storage temperatures for preserving viability and probiotic properties of lyophilized bacterial strains from chicken gut. PLoS ONE.

[140] Abdul Manan M. (2025). Progress in Probiotic Science: Prospects of Functional Probiotic-Based Foods and Beverages. Int. J. Food Sci..

[141] Gore A. Probiotics Market Report 2025 (Global Edition). https://www.cognitivemarketresearch.com/probiotics-market-report.

[142] Hill P., Muir J.G., Gibson P.R. (2017). Controversies and Recent Developments of the Low-FODMAP Diet. Gastroenterol. Hepatol..

[143] Murillo A.Z., Arévalo F.E., Jáuregui E.P. (2016). Diet low in fermentable oligosaccharides, disaccharides, monosaccharides and polyols (FODMAPs) in the treatment of irritable bowel syndrome: Indications and design. Endocrinol. Nutr. (Engl. Ed.).

[144] Fredua-Agyeman M., Larbi E.A. (2025). Inaccurate labelling practices in probiotic products: A regulatory shortfall in Accra, Ghana. PLoS ONE.

[145] Siegrist M., Shi J., Giusto A., Hartmann C. (2015). Worlds apart. Consumer acceptance of functional foods and beverages in Germany and China. Appetite.

[146] Chang M.Y., Chen H.S. (2022). Understanding Consumers’ Intentions to Purchase Clean Label Products: Evidence from Taiwan. Nutrients.

[147] Bimbo F., Bonanno A., Nocella G., Viscecchia R., Nardone G., De Devitiis B., Carlucci D. (2017). Consumers’ acceptance and preferences for nutrition-modified and functional dairy products: A systematic review. Appetite.

[148] Kraus A., Annunziata A., Vecchio R. (2017). Sociodemographic factors differentiating the consumer and the motivations for functional food consumption. J. Am. Coll. Nutr..

[149] Roman S., Sánchez-Siles L.M., Siegrist M. (2017). The importance of food naturalness for consumers: Results of a systematic review. Trends Food Sci. Technol..

[150] Rendahl J., Korp P., Ekström M.P., Berg C. (2017). Adolescents’ trust in food messages and their sources. Br. Food J..

[151] De Simone C. (2019). The unregulated probiotic market. Clin. Gastroenterol. Hepatol..

[152] Liang L., Yi Y., Lv Y., Qian J., Lei X., Zhang G. (2018). A comprehensive genome survey provides novel insights into bile salt hydrolase (BSH) in *Lactobacillaceae*. Molecules.

[153] Abdul Manan M. (2025). The role of probiotics in personalized therapeutics: Advances in gut microbe-driven interventions. Microbe.

[154] Abouelela M.E., Helmy Y.A. (2024). Next-Generation Probiotics as Novel Therapeutics for Improving Human Health: Current Trends and Future Perspectives. Microorganisms.

[155] McCoubrey L.E., Elbadawi M., Orlu M., Gaisford S., Basit A.W. (2021). Harnessing machine learning for development of microbiome therapeutics. Gut Microbes.

[156] Li P., Luo H., Ji B., Nielsen J. (2022). Machine learning for data integration in human gut microbiome. Microb. Cell Fact..

[157] Pedroza Matute S., Iyavoo S. (2023). Exploring the gut microbiota: Lifestyle choices, disease associations, and personal genomics. Front Nutr.

[158] Leuschner R.G., Robinson T.P., Hugas M., Cocconcelli P.S., Richard-Forget F., Klein G., Licht T.R., Nguyen-The C., Querol A., Richardson M. (2010). Qualified presumption of safety (QPS): A generic risk assessment approach for biological agents notified to the European Food Safety Authority (EFSA). Trends Food Sci. Technol..

[159] Koutsoumanis K., Allende A., Alvarez-Ordóñez A., Bolton D., Bover-Cid S., Chemaly M., Davies R., De Cesare A., Hilbert F., EFSA-BIOHAZ (2019). Update of the list of QPS-recommended biological agents intentionally added to food or feed as notified to EFSA 10: Suitability of taxonomic units notified to EFSA until March 2019. EFSA J..

[160] Huys G., Vancanneyt M., d’Haene K., Vankerckhoven V., Goossens H., Swings J. (2006). Accuracy of species identity of commercial bacterial cultures intended for probiotic or nutritional use. Res. Microbiol..

[161] Vankerckhoven V., Moreillon P., Piu S., Giddey M., Huys G., Vancanneyt M., Goossens H., Entenza J.M. (2007). Infectivity of *Lactobacillus rhamnosus* and *Lactobacillus paracasei* isolates in a rat model of experimental endocarditis. J. Med. Microbiol..

[162] Koirala S., Anal A.K. (2021). Probiotics-based foods and beverages as future foods and their overall safety and regulatory claims. Future Foods.

[163] Sanders M.E. (2009). How do we know when something called “probiotic” is really a probiotic? A guideline for consumers and health care professionals. Funct. Food Rev..

[164] Douillard F.P., Ribbera A., Kant R., Pietilä T.E., Järvinen H.M., Messing M., Randazzo C.L., Paulin L., Laine P., Ritari J. (2013). Comparative genomic and functional analysis of 100 Lactobacillus rhamnosus strains and their comparison with strain GG. PLoS Genet..

